# Supplementary figures and images for: A desynchrony mechanism between events triggers a compensatory delay during Caenorhabditis elegans development
Source: PLoS Biol. 2026 Jul 24;24(7):e3003867. doi: 10.1371/journal.pbio.3003867 (PMC13399346; doi:10.1371/journal.pbio.3003867)

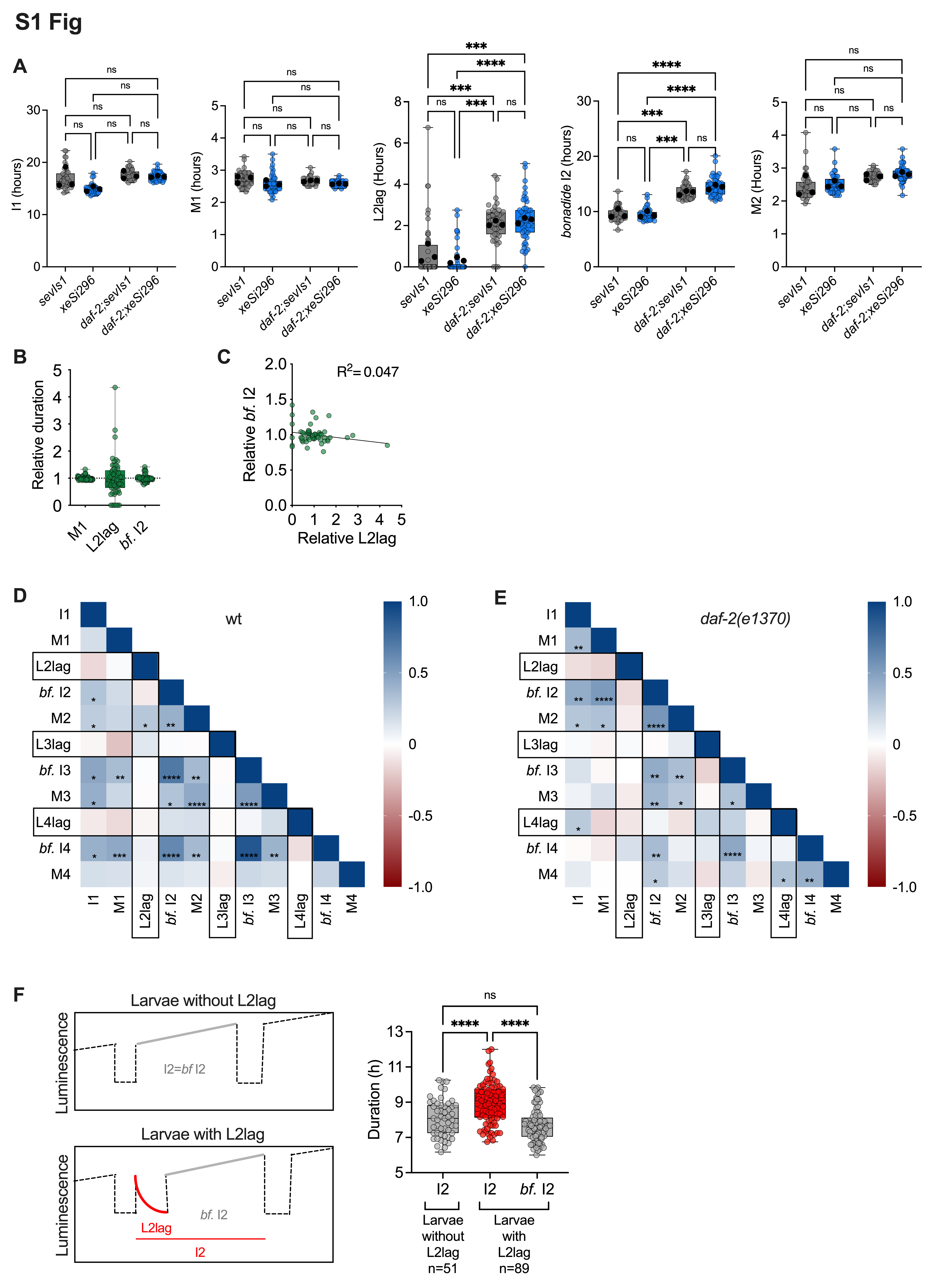

Supplement: S1 Fig — (A) Duration of I1, M1, L2Llag, bona fide I2 and M2 for wild type and daf-2 mutant with the luminescence reporters sevIs1 and xeSi296. (B) Duration of M1, L2lag and bona fide I2 relative to the average duration of each stage for the same larvae shown in Fig 1H. (C) Relative duration of L2lag and bf. I2 for each of the larvae in B. (D, E) Matrix heatmap of the correlation between the duration of all larval stages in 58 individual larvae of wildtype (D) and 57 individual daf-2(e1370) mutant larvae (E). (F) Diagrams showing the luminescence profile and durations analyzed, for wild-type larvae without and with L2lag (left), and duration of the corresponding periods (right). In A and F, we used One-way ANOVA followed by Tukey’s multiple comparisons to test differences between strains or developmental stages. Differences are marked as * p < 0.05, ** p < 0.01, *** p < 0.001 and **** p < 0.0001. The data represented in all plots can be found in S1 Data. (TIFF) [file pbio.3003867.s001.tiff]

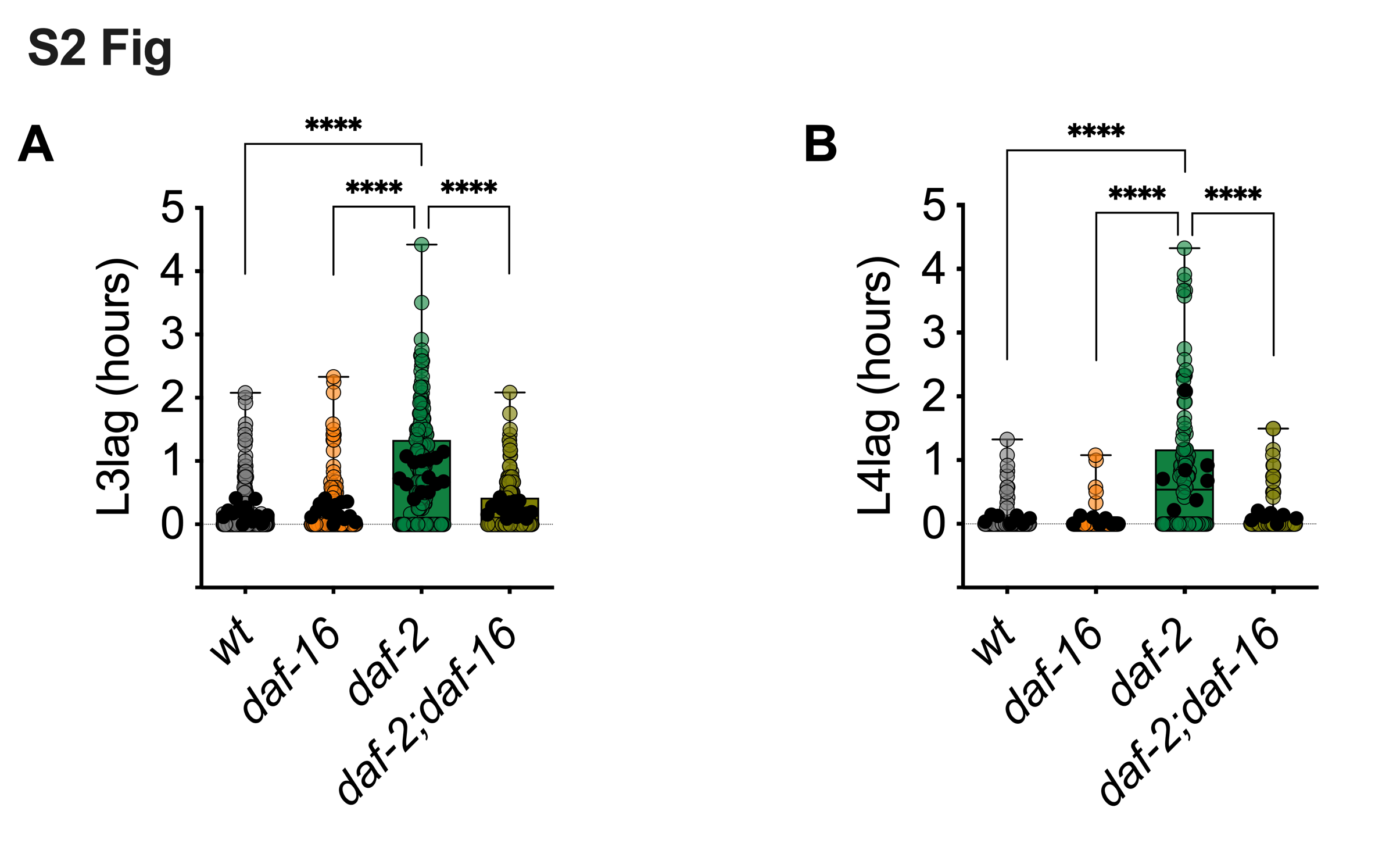

Supplement: S2 Fig — Duration of L3lag (A) and L4lag (B) for the wild type, daf-2, daf-16, and daf-2;daf-16. Colored circles show values for individual larvae and black dots show the average for each experiment. For both panels, we performed One-way ANOVA followed by Tukey’s multiple comparisons to test differences between all strains. Only significant differences are shown, **** p < 0.0001. The data represented in all plots can be found in S1 Data. (TIFF) [file pbio.3003867.s002.tiff]

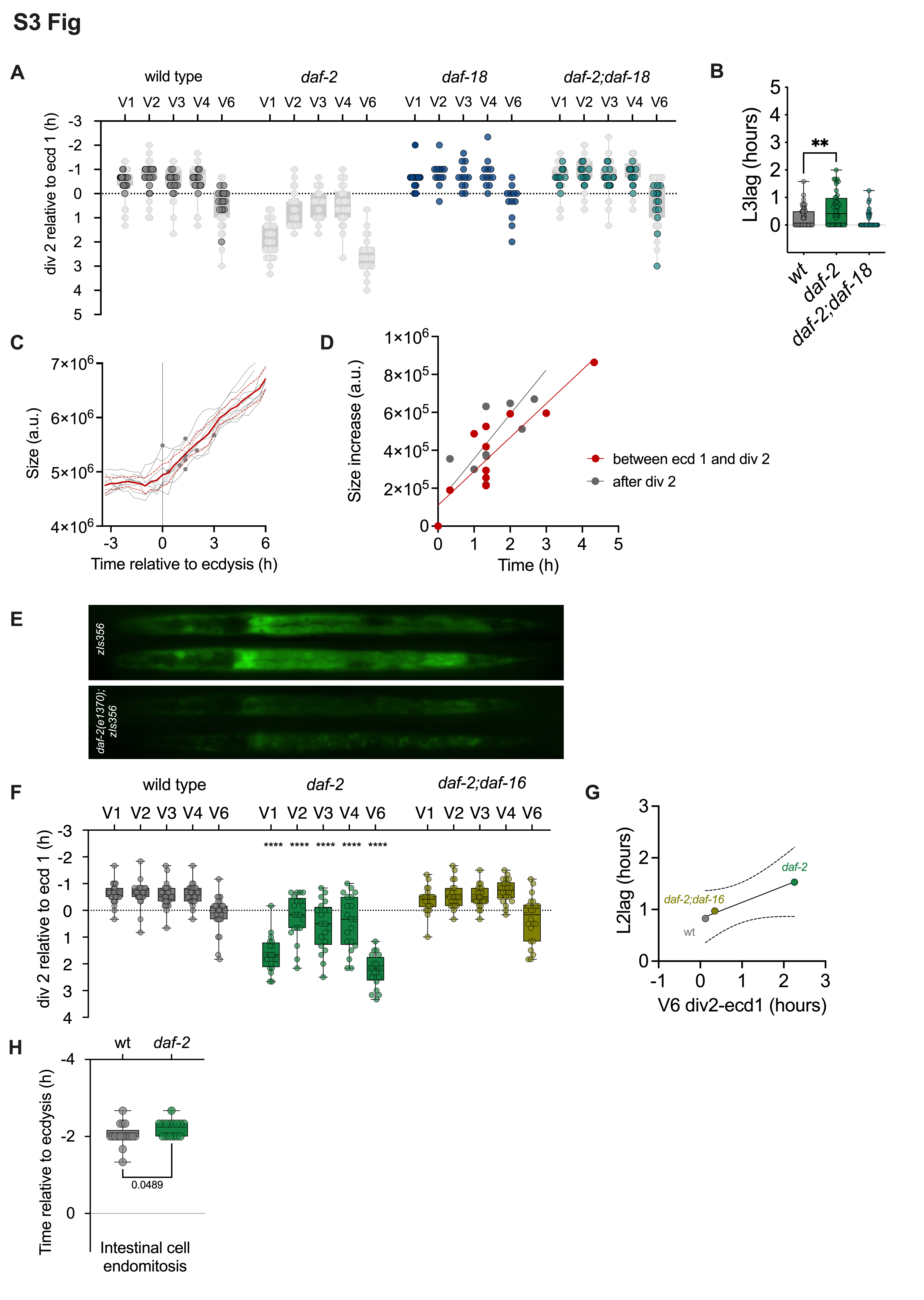

Supplement: S3 Fig — (A) Timing of seam cell division (div 2) relative to ecdysis (ecd 1) for the wild type, daf-18 and daf-2;daf-18. This data set is shown overlaid to the data presented in Fig 4D. (B) Duration of L3lag for the wild type, daf-2 and daf-2;daf-18. (C) Quantification of size relative to the timing of ecdysis. Gray lines show the values of individual larvae and red line shows the average (and 95% CI). Gray dots show the timing of seam cell division for each animal. (D) Size increase per time interval between ecd 1 and div 2 (red) and in a similar period of time after div 2. (E) Representative images of DAF-16::GFP in the wild type and daf-2(e1370) backgrounds. (F) Timing of seam cell division (div 2) relative to ecd 1 for the wild type, the daf-2 mutant, and the double mutant daf-2;daf-16. Colored circles show values for individual larvae. (G) Correlation between L2lag and the timing of V6 seam cell division relative to ecdysis (div 2-ecd 1). (H) Timing of intestinal cell endoreplication relative to ecdysis, for the wild type and daf-2. Statistics reflect the result of unpaired t test. For B and F, we performed One-way ANOVA followed by Tukey’s multiple comparisons to test differences between strains. Only significant differences are shown, ** p < 0.01, **** p < 0.0001. The data represented in all plots can be found in S1 Data. (TIFF) [file pbio.3003867.s003.tiff]

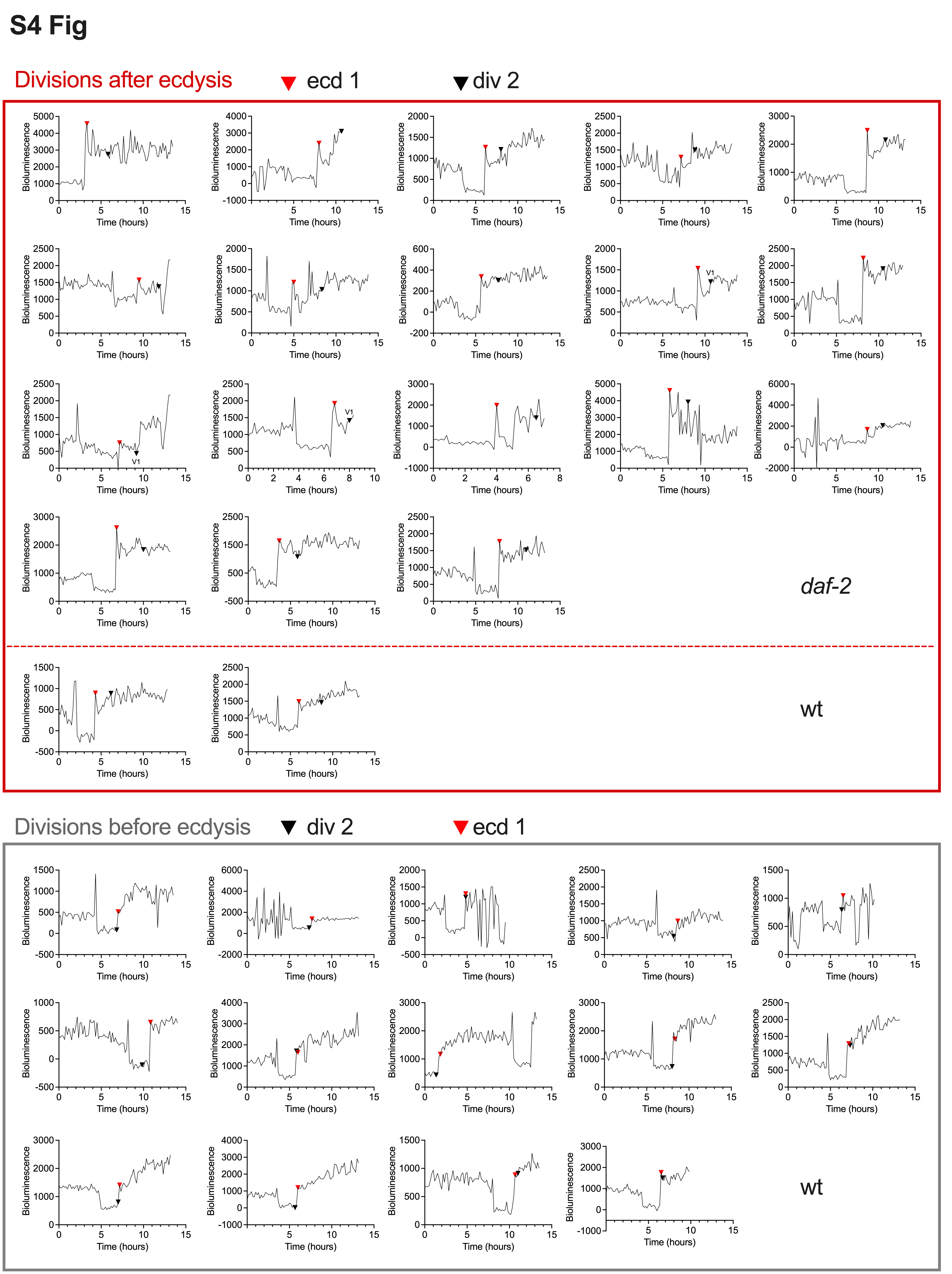

Supplement: S4 Fig — Luminescence profile from microscopy experiments for larvae categorized as seam cell divisions (div 2) ending after or before ecdysis (ecd 1). Red arrowheads signal ecdysis and black arrowheads mark V6 division, except when otherwise stated. The data represented in all plots can be found in S1 Data. (TIFF) [file pbio.3003867.s004.tiff]

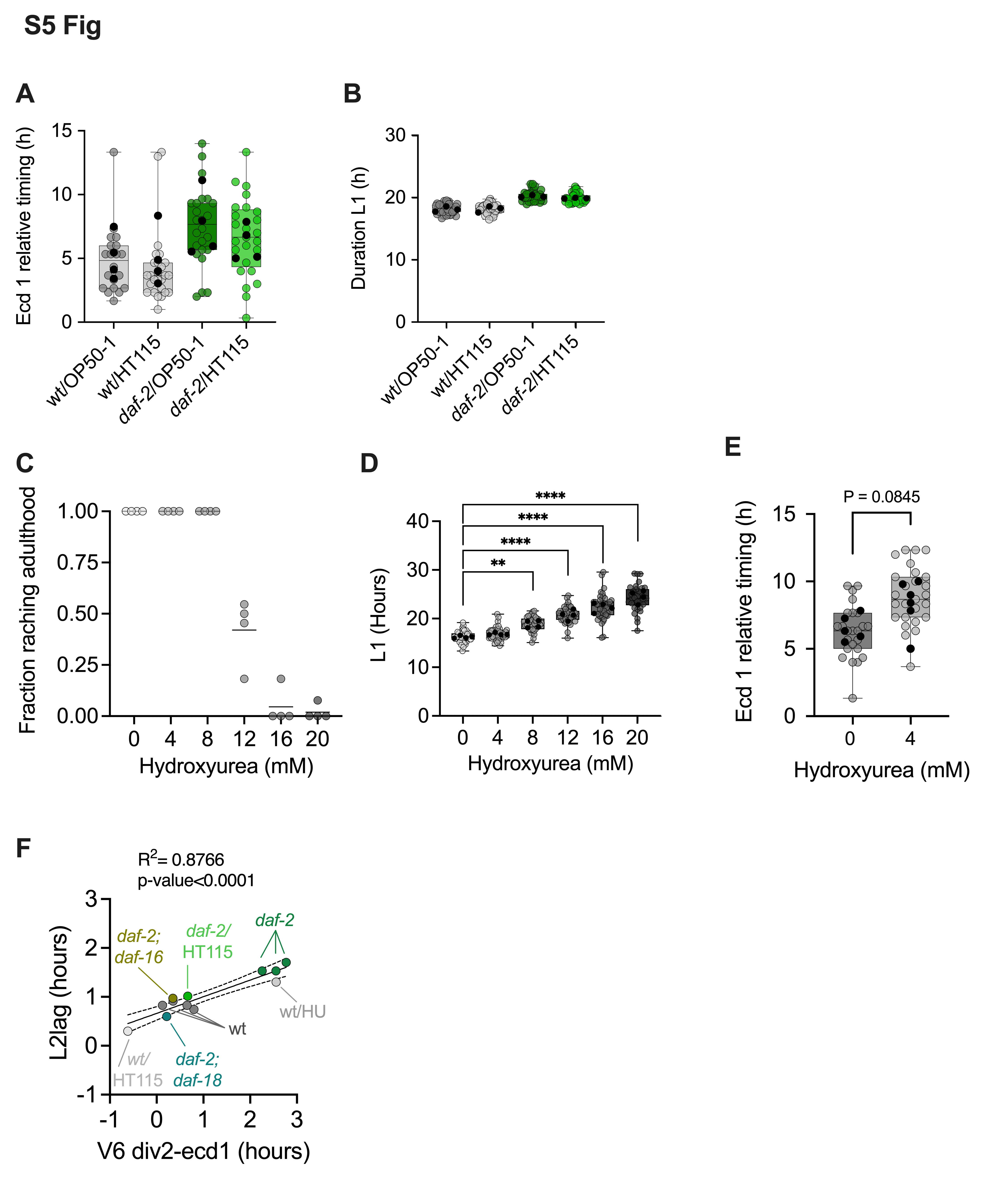

Supplement: S5 Fig — (A) Timing of ecdysis 1 (first release of the cuticle observed in time-lapse experiments) relative to the beginning of imaging in time-lapse experiments, for the wild type and daf-2(e1370) growing on OP50-1 or HT115. (B) Duration of the complete L1 stages, defined as the time from hatching to the end of the first molt, as inferred from the bioluminescence signal in a luminometer, for the same conditions as in A. (C) Fraction of animals that reach adulthood after treatment with different concentration of HU from L1, in four independent experiments. (D) Duration of the stage L1 after treatment with different concentrations of HU in four independent experiments. We performed One-way ANOVA followed by Dunnett’s multiple comparisons to test differences between each of the treatments and the untreated larvae. Differences are marked as ** p < 0.01, **** p < 0.0001. (E) Timing of ecdysis 1 (first release of the cuticle observed in time-lapse experiments) relative to the beginning of imaging in time-lapse experiments, for the wild type on 0- or 4-mM Hydroxyurea. Statistics reflect the result of unpaired t test. (F) Correlation between L2lag and the timing of V6 seam cell division relative to ecdysis (div 2-ecd 1) for all conditions and genotypes tested. Statistics reflect the result of unpaired t test. Differences are marked as ** p < 0.01, **** p < 0.0001. Gray or colored circles show values for individual larvae and black dots show the average of each experiment. The data represented in all plots can be found in S1 Data. (TIFF) [file pbio.3003867.s005.tiff]
