# Supplementary material for: A desynchrony mechanism between events triggers a compensatory delay during Caenorhabditis elegans development
Source: PLoS Biol. 2026 Jul 24;24(7):e3003867. doi: 10.1371/journal.pbio.3003867 (PMC13399346; doi:10.1371/journal.pbio.3003867)
Supplement: S1 Table — (PDF) [file pbio.3003867.s006.pdf]

S1 Table. List of strains

**Strains**	**Source**	**Code**
**Bacterial strains**		
E. coli OP50-1	*Caenorhabditis* Genetics Center	OP50-1
E. coli HT115(DE3) pL4440	Rual et al., 2004	
E. coli HT115(DE3) *lin-14* RNAi	Ahringer library	
E. coli HT115(DE3) *lin-28* RNAi	Ahringer library	
C. elegans		
sevIs1[Psur-5::luc+::gfp]X	Olmedo et al., 2020	MRS387
daf-2(e1370)III; sevIs1 [Psur-5::luc+::gfp]X	Olmedo et al., 2020	MRS434
xeSi296[eft-3p::luc::gfp::unc-54 30UTR, unc-119(+)] II	Meeuse et al., 2020	HW1939
daf-2(e1370)III;xeSi296[eft-3p::luc::gfp::unc-54 30UTR, unc-119(+)] II	This study	MOL906
xeSi440[dpy-9p::gfp::h2b::pest::unc-54 3'UTR; unc-119 +] II	Meeuse et al., 2023	HW2526
daf-2(e1370)III; xeSi440[dpy-9p::gfp::h2b::pest::unc-54 3'UTR; unc-119 +] II	This study	MOL908
daf-16(mu86)I; sevIs1 [Psur-5::luc+::gfp]X	Olmedo et al., 2020	MRS424
daf-2(e1370)III; daf-16(mu86)I; sevIs1[Psur-5::luc+::gfp]X	Olmedo et al., 2020	MOL56
daf-18(ok480) IV; sevIs1[Psur-5::luc+::gfp]X	This study	MOL267
daf-2(e1370); daf-18(ok480) IV x sevIs1[Psur-5::luc+::gfp]X	This study	MOL315
daf-16(mu86)I; daf-18(ok480)IV; sevIs1[Psur-5::luc+::gfp]X	This study	MOL433
daf-2(e1370)III; daf-16(mu86)I; daf-18(ok480)IV; sevIs1[Psur-5::luc+::gfp]X	This study	MOL434
lin-42(n1089)II; sevIs1[Psur-5::luc+::gfp]X	This study	MOL459
lin-42(ok2385)II; sevIs1[Psur-5::luc+::gfp]X	This study	MOL460
matIs38 [Pscm::CYB-1 DB-mCherry::unc-54 3' UTR; Pscm::NLS-GFP::tbb-2 3' UTR; Pmyo-2::GFP]	Dr. Matilde Galli Hubrecht Institute	GAL69
daf-2(e1370)III; matIs38 [Pscm::CYB-1 DB-mCherry::unc-54 3' UTR; Pscm::NLS-GFP::tbb-2 3' UTR; Pmyo-2::GFP]	This study	MOL185
daf-18(ok480)IV; matIs38 [Pscm::CYB-1 DB-mCherry::unc-54 3' UTR; Pscm::NLS-GFP::tbb-2 3' UTR; Pmyo-2::GFP]	This study	MOL895
daf-2(e1370); daf-18(ok480)IV; matIs38 [Pscm::CYB-1 DB-mCherry::unc-54 3' UTR; Pscm::NLS-GFP::tbb-2 3' UTR; Pmyo-2::GFP]	This study	MOL367
sevIs1[Psur-5::luc+::gfp]X; matIs38 [Pscm::CYB-1 DB-mCherry::unc-54 3' UTR; Pscm::NLS-GFP::tbb-2 3' UTR; Pmyo-2::GFP]	This study	MOL345
daf-2(e1370)III; sevIs1[Psur-5::luc+::gfp]X; matIs38 [Pscm::CYB-1 DB-mCherry::unc-54 3' UTR; Pscm::NLS-GFP::tbb-2 3' UTR; Pmyo-2::GFP]	This study	MOL344
daf-2(e1370)III; daf-16(mu86)I; sevIs1[Psur-5::luc+::gfp]X; matIs38 [Pscm::CYB-1 DB-mCherry::unc-54 3' UTR; Pscm::NLS-GFP::tbb-2 3' UTR; Pmyo-2::GFP]	This study	MOL435
matIs29 [Pges-1::CYB-1 DB-mCherry::unc-54 3' UTR; Pges-1::NLS-GFP::tbb-2 3' UTR; Pmyo-2::GFP]	Dr. Matilde Galli Hubrecht Institute	GAL45
daf-2(e1370)III;matIs29 [Pges-1::CYB-1 DB-mCherry::unc-54 3' UTR; Pges-1::NLS-GFP::tbb-2 3' UTR; Pmyo-2::GFP]	This study	MOL149
zIs356[Pdaf-16::daf-16a/b-gfp; rol-6] IV	CGC	TJ356
daf-2(e1370)III; zIs356[Pdaf-16::daf-16a/b::gfp; rol-6]	Olmedo et al., 2020	MOL72
