# Supplementary material for: A desynchrony mechanism between events triggers a compensatory delay during Caenorhabditis elegans development
Source: PLoS Biol. 2026 Jul 24;24(7):e3003867. doi: 10.1371/journal.pbio.3003867 (PMC13399346; doi:10.1371/journal.pbio.3003867)
Supplement: S2 Table — (PDF) [file pbio.3003867.s007.pdf]

S2 Table. Strains used, number of larvae, and experimental replicates.

	Label	Strain	n larvae	N replicates
**1E**	M1	MRS434	20	3
	L2lag		17	
	l2		15	
**1F**	M1	MRS434	17	5
	L2lag		16	
	l2		17	
**1G**	wt	MRS387	33	3
	daf-2	MRS434	30	
**1H**		MRS434	56	5
**1I-J**	wt	HW2526	12	4
	daf-2	MOL908	11	
**2B,C**	wt	MRS387	159	15
	daf-16	MRS424	192	
	daf-2	MRS434	126	
	daf-2;daf-16	MOL56	187	
**2D,E**	wt	MRS387	62	4
	daf-16	MRS424	49	
	daf-18	MOL267	54	
	daf-16;daf-18	MOL433	45	
	daf-2	MRS434	50	
	daf-2;daf-16	MOL56	42	
	daf-2;daf-18	MOL315	60	
	daf-2;daf-16;daf-18	MOL434	62	
**3B**	wt/pL4440	MRS387/ pL4440	33	3
	wt/*lin-14 RNAi*	MRS387/*lin-14* RNAi	34	
	wt/*lin-28 RNAi*	MRS387/*lin-28* RNAi	36	
	daf-2/pL4440	MRS434/ pL4440	29	
	daf-2/lin-14 RNAi	MRS434/*lin-14* RNAi	32	
	daf-2/lin-28 RNAi	MRS434/*lin-28* RNAi	33	
**3D**	wt	MRS387	112	5
	lin-42(n1089)	MOL459	109	
	lin-42(ok2385)	MOL460	58	
**4B**	*wt*	GAL69	15	4
	daf-2	MOL185	12	
	daf-2;daf-18	MOL367	16	
**4D**	*wt*	GAL69	37	9
	daf-2	MOL185	26	
	daf-2;daf-18	MOL367	34	
**4F**	*wt*	GAL69	21	5
	daf-2	MOL185	11	
	daf-2;daf-18	MOL367	14	
**5A**	L2lag	MOL344	11	5
	l2		10	
	l2 after L2lag		13	
**5B**	div 2 before ecd1	MOL345	14	9
	div 2 after ecd1	MOL345/MOL344	20 (2/18)	
**5C**	wt OP50-1	GAL69	20	4
	wt HT115		26	
	daf-2 OP50-1	MOL185	24	
	daf-2 HT115		24	
**5E**	wt OP50-1	MRS387/OP50-1	40	3
	wt HT115	MRS387/HT115	36	
	daf-2 OP50-1	MRS434/OP50-1	41	
	daf-2 HT115	MRS434/HT115	38	

**5F**	0 mM HU	GAL69	23	6
	4 mM HU		21	
**5H**	0 mM HU	MRS387	44	4
	4 mM HU		43	
**6A**	12 ºC	MRS387	38	3
	16 ºC		61	3
	20 ºC		40	3
	22 ºC		38	3
**6B,C**	20 ºC	GAL69	15	4
	22.5 ºC		15	4
**6D**	12 ºC	MRS434	46	3
	16 ºC		61	3
	20 ºC		39	3
	22 ºC		38	3
**6E,F**	20 ºC	MOL185	13	4
	22.5 ºC		18	4
