# Supplementary material for: A desynchrony mechanism between events triggers a compensatory delay during Caenorhabditis elegans development
Source: PLoS Biol. 2026 Jul 24;24(7):e3003867. doi: 10.1371/journal.pbio.3003867 (PMC13399346; doi:10.1371/journal.pbio.3003867)
Supplement: Attachment — Submitted filename: Response to reviewers.pdf [file pbio.3003867.s012.pdf]

Romero-Expósito et al., (PBIOLOGY-D-25-02981R1) Response to Reviewers

We are grateful to the reviewers for their thorough evaluation of our work. Several of their comments could be addressed immediately based on existing literature and data already obtained in our laboratory. Other comments motivated us to perform additional experiments, which we have now completed and summarized below. Altogether, this feedback has strengthened the clarity of the manuscript and further supported the evidence presented in our study.

After this summary, we include a point-by-point response to all reviewers' comments.

1) Comprehensive characterization of the L2Lag state.
In response to concerns raised by all three reviewers, we performed an extensive set of new experiments that substantially deepen the characterization of the L2Lag. These analyses show that larvae in L2Lag maintain stable luciferase protein levels, display no morphological abnormalities, and retain both movement and pumping activity, although with distinct dynamics compared to M1 and I2. We further demonstrate that larval growth continues after ecdysis, independently of the state of seam cell division. Together, these findings rule out the possibility that the reduced bioluminescence signal arises from decreased luciferase expression or failure to ingest luciferin. Additional analyses confirm that this pause time is added to the *bonafide* intermolt duration. This deeper characterization thus validates L2Lag as a real biological state, not a measurement artifact.

2) Oscillation for the entire L2 period with the reporter *dpy-9*p::GFP.
Addressing the reviewers' requests, we now provide a more complete assessment of oscillatory gene expression throughout the entire L2 stage. To overcome the limitations of the original F11E6.3 reporter, we repeated the experiment using *dpy-9*p::GFP, a more conventional molting oscillator expressed in a different oscillatory phase. The time-lapse image acquisition now spans the entire L2 stage, enabling stage-rescaled representations of the signal. *daf-2* mutants consistently show a delayed onset of oscillatory gene expression, demonstrating that the effect is not explained by mere global scaling of a longer L2 stage.

3) Correlation between L2lag and seam division timing across all conditions tested.
To directly assess the proposed model that L2Lag emerges when seam cell divisions become desynchronized from ecdysis, we expanded our correlation analyses across all genotypes and perturbations, including *daf-2*, *daf-16*, *daf-18*, HT115 feeding and HU treatment. Across conditions, the timing of seam cell divisions relative to ecdysis consistently correlates with the duration of L2Lag. While this correlation does not imply strict causal dependence on seam cell division completion, it strongly supports the idea that seam cell timing serves as a readout of a broader developmental desynchrony that the organism resolves via L2Lag.

Point-by-point response to reviewers:

Rev. 1:

In this manuscript, Romero-Exposito and colleagues describe a new transient larval pause state in C. elegans post-embryonic development. This state occurs between the L1 molt and the L2 intermolt, therefore termed L2Lag. Although this pause is initially identified in daf-2 mutants that display slow development, the L2Lag state is also detectable in wild-type animals grown under normal conditions, albeit at lower rates. The authors propose that this pause allows distinct developmental events to synchronize before initializing the next larval stage. In particular, they observe that the L2Lag tends to occur when seam cell divisions are delayed relative to ecdysis. Although the underlying cause and physiology of the L2Lag could be better described, this body of work represents an interesting finding that would be of broad interest to the developmental biology community and should be suitable for publication after minor revisions.

Major comments:

1. The L2Lag is a feature of the luminometry assay. It would be extremely important to validate its existence with an alternative method. The experiments with the F11E6.3 reporter in Fig1h-j represent a good initial step towards this goal. However, it is currently not possible to assess whether the delay in F11E6.3 induction observed in some worms by fluorescence microscopy corresponds to the L2Lag detected by luminometry or not. The authors should perform dual luminometry and fluorescence microscopy in microchambers using the F11E6.3 reporter. This should be technically feasible as evidenced by Fig5b. Such cross-validation would eliminate any doubt that the L2Lag is an artifact of the luminometry assay.

We agree with the reviewer that this would be a sensible experiment to show that the delay in F11E6.3 induction observed in some worms by fluorescence microscopy corresponds to the L2Lag.

However, we would like to remind the evidence that oppose the idea that L2Lag is an artifact of the luminometry assay:

- We have shown that the L2lag analyzed by luminometry is connected to the delay in seam cell division relative to ecdysis. This delay is measured in a completely different assay (time-lapse imaging following seam cell by fluorescence), in strains that don´t even carry the luminescence reporter (Fig. 4, 5). Actually, the two features should correlation between genotypes and throughout the conditions tested (now shown in Fig. 4E, 5I and S5F).

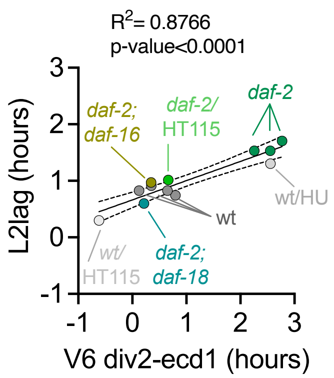

- What is key is that L2lag is a period of time that is *added* to the (*bonafide)* intermolt, as opposed to a feature that happens to be identified during the duration of the regular intermolt due to the luminescence signal.

We have now added new analysis to reinforce this idea (Fig. S1F), based on the duration of the L2lag and *bf*. I2. Importantly, we performed this analysis on wild-type larvae, to show how this is independent of possible *daf-2* related phenotypes as reduced feeding.

For larva that present L2lag, if we represent the duration of the complete intermolt, strictly considering the time between molts, its duration is significantly higher than that of larvae that do not enter this stage. By subtracting the duration of the L2lags, we obtain a *bonafide* I2 intermolt duration that is statistically not different from the average I2.

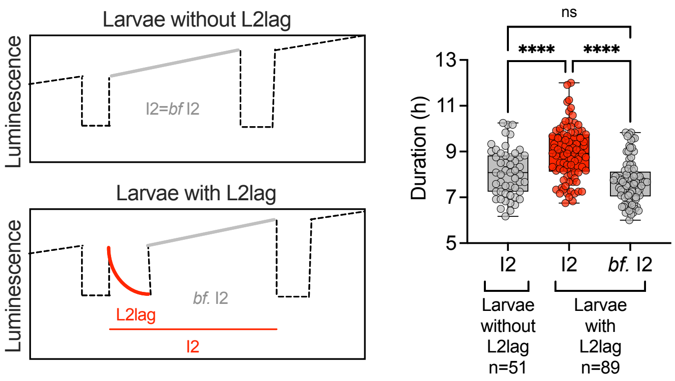

- Additionally, we commonly synchronize larvae of N2 (wild type) and observe that in plates with around 1000 animals there are usually a few that look significantly delayed. We have quantified that for N2(wild type) and CB1370(*daf-2*), observing around 1-2% and 5-8% delayed animals respectively. These numbers are low compared to the levels of L2lag but in this case the visual inspection only allows to detect those with strong delays (possibly very long L2lag).

Our observations from the luminometry assay explain that these are not just "overall slow" larvae. Importantly, only a time-resolved, single-larva method as the one we apply allows observing this type of features of development. As such, this featured of development has passed unnoticed for a long time.

Regarding the proposed experiment:

To be able to connect the delay in F11E6.3 with L2lag, we would need to be able to monitor bioluminescence and fluorescence in the same larva.

We tried this before for the experiment shown in Fig. 5B, to connect luminescence with fluorescence (in that case to assess seam cell divisions). The challenge for the proposed experiment, would be the same we had then as it concerns the imaging of luminescence. This

challenge is technical, as the intensity of bioluminescence signal emitted by single developing larvae is too low to be imaged. Imaging of luminescence cannot be detected by standard CCD or CMOS cameras and requires special high-sensitivity CCD sensors that are cooled to -100°C (instead of -10°C as standard CCD cameras) and have extremely low noise readout. For this reason, we attempted this at the laboratory of Prof. Merrow, that has such a setup (iKon-M 934 CCD camera).

Even with this setup, we obtained a very noisy luminescence signal. For this experiment we needed to apply an exposure time of 1 minute, while in the luminometer each well is read for 5 seconds (Please also note that we use high sensitivity luminometers for our assay with single larvae). Furthermore, time resolution is reduced from 5 to 10 min-intervals due to acquisition timing.

The plots below show two examples of quantification of the signal in the complete well (as it is not possible to delimit the outline of the moving larvae due to the 1-min exposure time) and the background, and the results from the difference between both values.

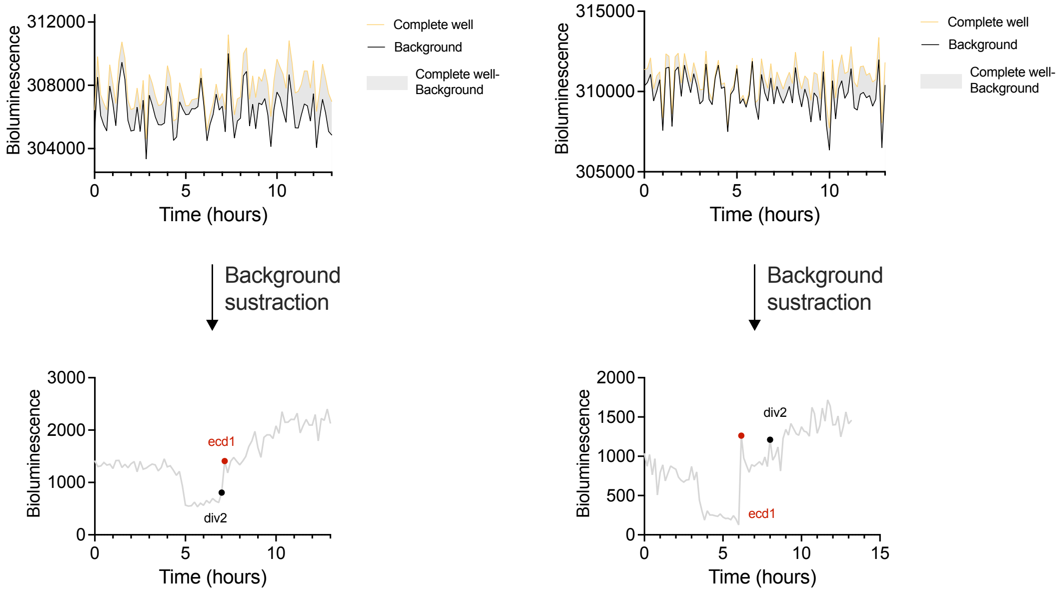

While the molt is clearly visible, the L2lag is not always as clear for individual larvae, making difficult to correlate divisions (or possible F11E6.3 expression patterns) with an increase in the signal.

For those experiments, we could stablish categories with larvae to continue with the analysis:

A) Last div2 (V6 seam cell) occurs before ecd1
B) Last div2 (V6 seam cell) occurs after ecd1

However, this would not be possible for F11E6.3 expression patterns, as the gradual increase of signal would not allow making categories.

With all this in mind, even for a test, we would need to make new constructs with F11E6.3 promoter fused to a different fluorophore, as the luciferase strain also contains GFP to control for expression levels of the transgene.

Nevertheless, in this version of the manuscript we present new data on the expression of the molting reporter *dpy-9*, this time over the complete L2 stage, so that we can present the signal rescaled to the duration of L2 for each individual larva (Fig. 1I, J). We think this new data shows more convincedly the delay expected in the *daf-2* mutant.

2. In terms of the worm physiology, what is happening during the L2Lag that prevents luminescence from rising? The assumption would be that the worms are not eating much during the L2Lag. The authors should determine if this is indeed the case and identify its underlying cause.
Is the cuticular plug still blocking the mouth during the L2Lag? Or is this a behavioral phenotype caused by by low pharyngeal pumping rates? More generally, what do the larvae look like during this state? It would be nice to see a few pictures of animals in L2Lag in the supplement. Do the larvae grow volumetrically during L2Lag or not?

In response to this comment, as well as to that of reviewers 2 and 3, we have worked on further characterizations of the L2lag period.

- We have discarded the possibility of reduced luciferase expression being responsible for the reduced luminescence signal. We have measured GFP levels at different moments, specifically M1, L2lag and I2, using the same approach as in Fig. 5A. In this experiment, we observe similar levels of GFP in all categories, concluding that the decrease in luminescence during L2lag signal does not come from a reduction in expression of LUC::GFP. The results have been included as a further characterization of L2lag in Fig. 1E.

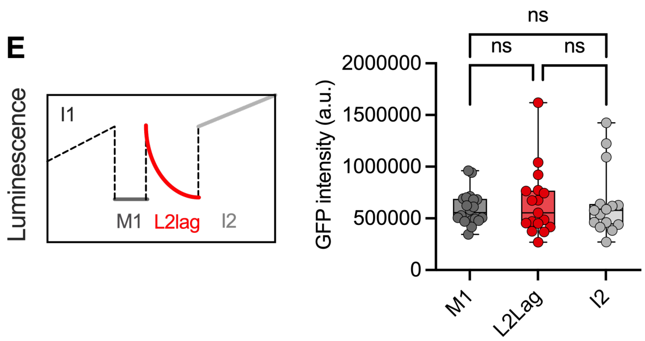

- Throughout these experiments, we could visualize larvae in the L2lag stage. These larvae showed no apparent morphological differences relative to the other stage and did not present any cuticular remnants. We have included this information in the text.

- We have checked the movement of L2lags and compare it to that of larvae in M1 and I2. Larvae in L2lag have levels of movement intermediate between M1 and I2. This result is included as Fig. 1 F.

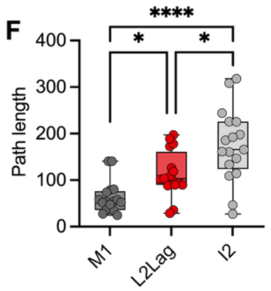

- We could also confirm that larvae pump after ecdysis during the period of seam cell desynchrony and low luminescence signal, but these experiments do not allow measure the rate of pumping.

- Additionally, we have measured growth over the period between ecdysis and seam cell divisions. We observed that growth continues during this period, and it happens to a similar rate as the growth after divisions. This information is included in the manuscript as follows:

"In these experiments, we could observe pumping after ecdysis, independently of the state of seam cell division. Furthermore, we measured the length of the larvae before and after ecdysis, while monitoring seam cell division, and observed that larvae grew at a constant rate after ecdysis, independently of the timing of cell divisions (Fig. S3C). For the same group of larvae, the growth rate was similar between ecdysis and div 2, and after div 2 (Fig. S3D)."

In summary, during L2lag, the luciferase levels are constant, the larvae are moving and pumping, and they keep growing. With this information, as the reviewer suggest, the assumption would be that the worms are eating, but probably less than larvae in I2.

3. The data shown in Fig. 4 is beautiful and very convincing that there is a correlation between desynchrony of seam cell divisions relative to ecdysis and the L2Lag state. The authors then argue that the partial suppression of the L2Lag in daf-2;daf-16 double mutants compared to daf-2;daf-18 is caused by the residual delay of V6 cell division in the daf-16 background in Fig.S2d. This argument appears in lines 236-239. However, both wild-type animals and daf-2;daf-18 mutants show similar levels of V6 division delay as daf-2;daf-16 mutants. Hence, this cannot possibly be the cause of the partial suppression of L2Lag by daf-16. The authors should remove this argument from the manuscript and simply acknowledge they cannot explain why daf-16 only partially suppresses the L2Lag.

We agree with the reviewer that the different between the suppression by *daf-18* and *daf-16* are subtle. In the current version of the manuscript, we show new data that includes the wild-type strain in Fig. S3F, to allow comparison of the *daf-2;daf-16* with this control. Unlike *daf-2;daf-18*, the double mutant *daf-2; daf-16* shows a later timing of divisions than the wild type Fig. S3G. We have provided these details in the text but, as the differences between the wild type and *daf-2;daf-16* are not statistically significant, we have removed the argument on the partial rescue.

4. The authors should also acknowledge that the perturbations they use to accelerate or delay seam cell divisions (HT115, HU) are non-specific and could be impacting many other aspects of animal development beyond their intended target. The language regarding causality should therefore be toned down in light of the limitations of the perturbations used.

The reviewer is right that the perturbations we used are non-specific, so we have now stated that in the text. However, it is important to remind that we check their impact on divisions and ecdysis, confirming that the relationship between them changes in the expected direction. The specific changes that we aimed for are obtained with the treatments, besides possibly affecting other processes.

-Changes to manuscript:

We modified the sentence on HT115 to:
"While feeding with HT115 might impact additional processes, we could confirm that HT115 significantly advanced seam cells divisions compared to OP50-1, in both the wild type and *daf-2(e1370)* (Fig. 5C), without impacting the timing of ecdysis (Fig. S5A)."

We modified the sentence on HU treatment to:
"As before, treatment with HU might impact other processes. To confirm the impact of HU on the timing of seam cell divisions relative to ecdysis, we followed these two features on the larvae treated with 4 mM HU".

Minor comments:
1. The authors should justify their choice of the F11E6.3 reporter for molting oscillations. This oscillator is unusual in that it only peaks in the L2-L4 stages, but not in L1. Why not use other reporters that peak in all larval stages, therefore allowing better detection of pauses in oscillatory dynamics, like dpy-9 or qua-1?

2. For the F11E6.3, why is a plateau observed after induction instead of the expected oscillatory profile?

We reply to these two comments together, as they are closely related.

At the time, we chose F11E6.3 because of the timing of signal increase corresponds to that of the beginning of intermolts, according to previous literature (https://pubmed.ncbi.nlm.nih.gov/32687264/ , Fig. S4). From this publication is also clear how F11E6.3p::GFP increases shortly after the first molt, peaks mid-L2 and then decreases before the next molt. This was not directly visible in the figure we showed in the paper because we focused on the fraction of the process when the signal increases, so that we could calculate the timing of 50% signal increase to use it as a marked. The complete dataset we acquired for the first version of the manuscript is shown below. In this graph is clear that the signal decreases at later times of development.

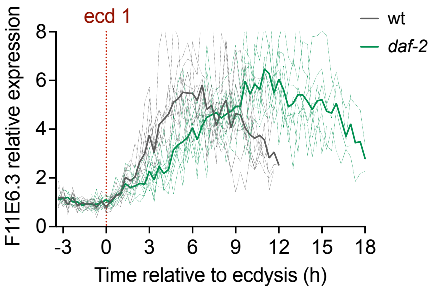

It is important that, as shown in the original publication, the signal never reaches the lower levels found in the previous state. Instead, the signal during the troughs increases gradually over development.

Indeed, the dynamics we found in the wild type for the F11E6.3 reporter is similar to that in the original publication. In the picture below, our sampling time is marked in red over the original figure.

(https://pubmed.ncbi.nlm.nih.gov/32687264/ , Fig. S4A-B).

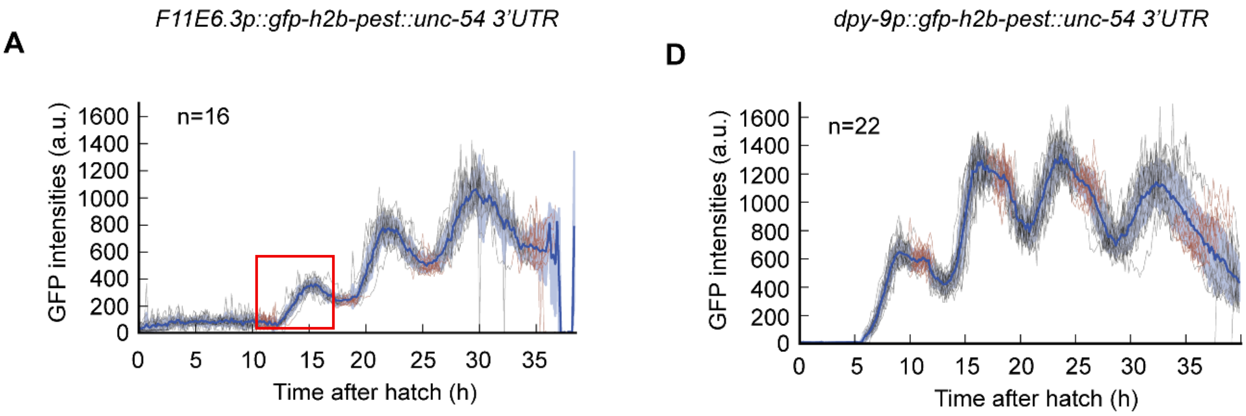

Nevertheless, the original data we acquired did not include the complete larval stage (Ecdysis 1 to ecdysis 2) for all worms. Furthermore, the reviewer raised the point that the F11E6.3 reporter is unusual in that its expression does not peak in L1. Additionally, reviewer 2 suggested that from the former analysis in Fig. 1H-J, it seemed difficult to exclude an alternative scenario where the signal merely scales with a longer L2 in *daf-2* mutants.

To overcome these issues, we have now repeated these experiments using the reporter *dpy-*9p::GFP (HW2526) in a wild-type and *daf-2* backgrounds. In this occasion, we have acquired images from the end of L1 to the beginning of L3, therefore including the complete L2 stage. This way, we could rescale the *dpy-*9p::GFP signal to the duration of the L2 stage in each individual larva. In these experiments, the GFP signal increase in *daf-2* shows a delay compared to the wild type.
The results from these experiments are shown in Fig. 1J.

- Changes to manuscript:
"We performed time-lapse microscopy of single larvae contained in 275x275x30 mm hydrogel microchambers [16] to monitor the expression of *dpy-9* in the wild type and *daf-2* mutants during the complete L2 stage. On average, the increase of *dpy-9* signal was delayed in the *daf-2* mutant (Fig. 1I). However, as the duration of the L2 stage is different between the wild type and the *daf-2* mutant, we plotted the data rescaled to the duration of each individual larva. Even after rescaling, we found that the signal increased later in *daf-2* mutants (Fig. 1J). This data supports the existence of delay in the expression of oscillatory gene expression at the beginning of L2 in *daf-2* mutants."

3. The section describing the epistasis analysis between daf-2, daf-16, and daf-18 is written in a confusing way. Especially the sentence in lines 166-168. The authors should clarify what they mean using simpler language.

The reviewer is right that the text was excessively complicated. Additionally, based on the subtle differences in suppression between *daf-16* and *daf-18* in this figure and Fig. 4, we have now focused only on that idea that both reduce L2lag in the daf-2 background, only *daf-18* reduces further than *daf-16*. We have simplified the paragraph as follows:

"For the regulation of L2lag, the *daf-18* mutant reduces the duration measured in the wild-type background [*daf-2 (+)*] (wild type vs. *daf-18* p-value=0.0018) and the *daf-2* mutant background [*daf-2 (-)*] (Fig. 2E). In the *daf-2* mutant background, suppression by *daf-18* is stronger than that by *daf-16* (Fig. 2E)."

4. The variability of the L2Lag duration seems to decrease in some genetic backgrounds (e.g. daf-18). It would be nice for the authors to perform CV analysis as in Fig. 1f for the other conditions tested.

We have calculated CV for L2lag and *bonafide* l2 for all genotypes related to insulin signaling. As in Fig. 1, the conclusion is that L2lag is highly variable compared to other larval stages. The variability might seem lower in *daf-18* because the levels of L2lag are low, but upon calculation of CV this is clearly not the case. We did not include this in the manuscript at this time, but we could do so if necessary.

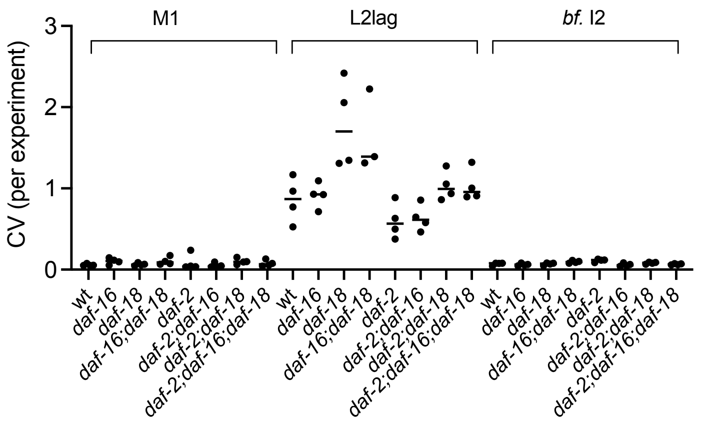

5. The lin-42(ok2385) mutant seemingly shows the strongest suppression of L2Lag among all the genotypes tested. If possible, it would be interesting to know if daf-2 mutation can still lengthen the L2Lag in lin-42(ok2385) background? This is an optional experiment.

We have performed the suggested cross and the experiments, but the *daf-2(e1370);lin-42(ok2385)* mutants show important developmental defects that drastically impact the reliability of the luminometry assay.

These are some examples of the luminometry traces of the *daf-2;lin-42* mutant:

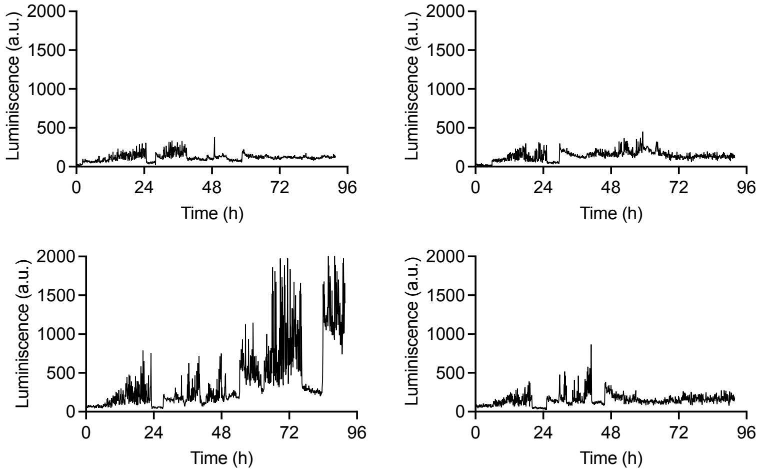

This contrasts with the clear pattern of luminescence obtained in rest of conditions tested before. Both automated analysis and visual inspection of the luminescence pattern of the double mutants fails to detect the duration of the larval stages. While the interaction of *lin-42* and *daf-2* is interesting and has partially been investigated before (https://pmc.ncbi.nlm.nih.gov/articles/PMC2947761/), we think the difficulty to analyze molting patterns limits the possibility to extract trustable conclusions about their combined impact on L2lag.

6. In Fig. 5a, it is unclear when exactly the animals were collected for seam cell division analysis. Can the authors please add an arrow noting the time of collection?

The animals were collected within 0.5-1 hour from the previous transition, that is from the end of M1 for animals in the categories "L2lag" and "I2", and from the end of L2lag for the larvae in the category" I2 after L2lag".
We have added arrows to mark the approximate time of collection.
Additionally, we have completed the information on how the samples are collected.

Changes to manuscript:

"We collected animals that, at the time of collection, were within 0.5-1 hour from the previous transition, that is, from the end of M1 for animals in the categories "L2lag" and "I2", and from the end of L2lag for the larvae in the category "I2 after L2lag."

7. For Fig.5b, tracks from individual animals should be separately shown in the supplement (i.e. one graph per animal) with the timing of seam cell divisions overlayed on the luminescence profile. This will allow readers to assess the correlation on a per-animal basis.

As explain before in the response to Major point 1, the bioluminescence signal acquired in the microscope is very noisy and it is difficult to clearly detect the L2lags, especially the end of it. This is way we averaged the signal for Fig. 5B. Nevertheless, we have now plotted in Fig. S3 the bioluminescence signal of all the animals independently, with overlayed marks showing the time of ecd 1 and div 2.

8. Fig. S3a shows relative timing of ecdysis - relative to what?

We have used the term Ecd 1 "relative timing" as this is not the timing of ecd1 from hatching but from the beginning of the time-lapse imaging. For this experiment, we synchronized larvae and placed them in chambers, but started imaging after 20-22 hours. We have now clarified this in the figure legend.

Note: Former Fig S3 is now S5

"Figure S5. (Relative to figure 5). (A) Timing of ecdysis 1 (first release of the cuticle observed in time-lapse experiments) relative to the beginning of imaging in time-lapse experiments, for the wild type and *daf-2(e1370)* growing on OP50-1 or HT115."

We have proceeded in the same way for Fig. S5E.

9. Fig. S3e, there does seem to be a trend towards delayed ecdysis with HU treatment. What is the p-value? Are there outliers driving the lack of significant difference?

The reviewer is right that there is a trend towards delay ecdysis. The values do not show outliers, and the p-value is 0.0845, as we have now indicated in the corresponding panel. Nevertheless, this ecdysis timing is only approximate, as in this specific experiment we did not image from hatching. Times refer to the beginning of imaging and thus labelled as Ecdysis "relative" timing. The complete during from hatching to ecdysis is better assessed in the luminometer (now Fig. S5D).

Nevertheless, the key idea is that this condition leads to increased desynchrony between ecdysis and divisions (independently of having no or little effect in the timing of ecdysis).

Rev. 2:
In their study, Romero-Exposito and colleagues investigate mechanisms affecting the synchrony between different developmental events in C. elegans larvae, specifically focusing on the divisions of hypodermal seam cells and molting (ecdysis). Using a previously developed luciferase-based assay, where lethargus/molt can be distinguished from the animal's growth/intermolt phase by the absence/presence of luminescent signal, they discover that some animals exhibit an in-between pattern, with a period of intermediate luminescence levels following ecdysis. This pattern, termed L2lag, is particularly pronounced and penetrant when examining the beginning of L2, and in daf-2 mutant animals, which are impaired in insulin/IGF1 signaling. To elucidate the L2lag mechanism, the authors explored the contribution of different components of the insulin/IGF1 signaling pathway, namely the transcription factor DAF-16 and the phosphatase DAF-18. By combining luminescence analysis, imaging, genetics and chemical perturbations, Romero-Exposito et al. develop a model in which cell division and ecdysis are controlled by distinct timers, responding differently to perturbations such as daf-2 mutation. To resynchronize them after perturbation, an undefined mechanism comes into play and initiates the L2lag, pausing the molting timer, thus preventing the amplification of the desynchrony over the course of the development.

The idea of distinct timers that achieve synchronization by putting one "on hold" is appealing and the authors' approaches are well-chosen and cutting-edge. The question is interesting and the approaches solid, although we find some of the claims over-interpreted, requiring further evidence and/or qualification. Specific points are listed below, but to summarize briefly: to qualify as a "state", we think that L2lag would deserve a more thorough characterization. Moreover, it remains puzzling why the effect appears largely limited to L2, especially in wild-type animals. This appears to contrast with the authors' claim of having demonstrated general modularity of C. elegans (larval) development. In addition, for daf-2, it is unclear how specific the effect is to the mutant allele employed. Finally, it seems to us that incomplete seam cell division is insufficient to explain the lag, several conditions appear to exhibit major differences in division timing but rather modest effects on L2lag occurrence or duration; this point would deserve clarification in the discussion.

We thank the reviewer for the detailed analysis, but we would like to remind that the lags also occur at the beginning of L3 and L4, as shown in Fig. 1G, Fig. S1D, E, and in new Fig. S2A,B.

Major points (for points involving experiments, we indicate whether essential, strongly recommended, or optional):

1. ESSENTIAL: The L2lag phase requires better characterization, at this point, it is not clear whether this is a developmental or a behavioral phenomenon. Do the worms still have a plug? Are they pumping? Are they moving?

In response to this comment, as well as to that of reviewers 2 and 3, we have worked on further characterizations of the L2lag period.

- We have discarded the possibility of reduced luciferase expression being responsible for the reduced luminescence signal. We have measured GFP levels at different moments, specifically M1, L2lag and I2, using the same approach as in Fig. 5A. In this experiment, we observe similar levels of GFP in all categories, concluding that the decrease in luminescence during L2lag signal does not come from a reduction in expression of LUC::GFP. The results have been included as a further characterization of L2lag in Fig. 1E.

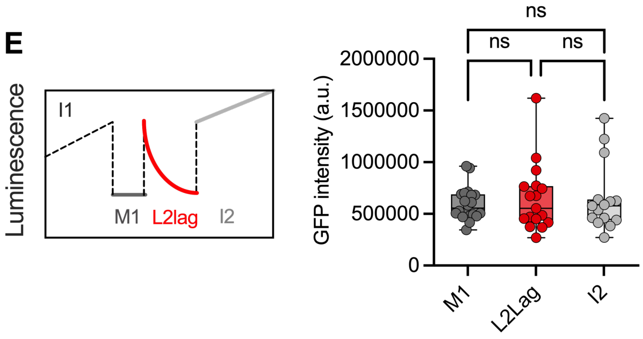

- Throughout these experiments, we could visualize larvae in the L2lag stage. These larvae showed no apparent morphological differences relative to the other stage and did not present any cuticular remnants. We have included this information in the text.

- We have checked the movement of L2lags and compare it to that of larvae in M1 and I2. Larvae in L2lag have levels of movement intermediate between M1 and I2. This result is included as Fig. 1 F.

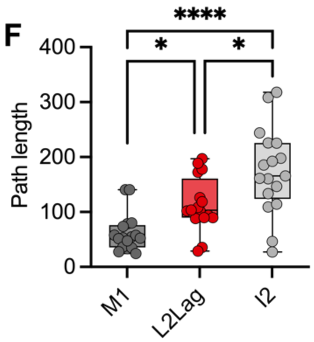

- We could also confirm that larvae pump after ecdysis during the period of seam cell desynchrony and low luminescence signal, but these experiments do not allow measure the rate of pumping.

- Additionally, we have measured growth over the period between ecdysis and seam cell divisions. We observed that growth continues during this period, and it happens to a similar rate as the growth after divisions. This information is included in the manuscript as follows:

"In these experiments, we could observe pumping after ecdysis, independently of the state of seam cell division. Furthermore, we measured the length of the larvae before and after ecdysis, while monitoring seam cell division, and observed that larvae grew at a constant rate after ecdysis, independently of the timing of cell divisions (Fig. S3C). For the same group of larvae, the growth rate was similar between ecdysis and div 2, and after div 2 (Fig. S3D)."

In summary, during L2lag, the luciferase levels are constant, the larvae are moving and pumping, and they keep growing. With this information, as the reviewer suggest, the assumption would be that the worms are eating, but probably less than larvae in l2.

RECOMMENDED: Have the authors looked at other developmental events that can be timed; e.g., is Q-cell migration normal in daf-2?

We thank the reviewer for the recommendation to analyze other cell types. While the Q-cell migration is an interesting process during L1, this process starts almost immediately after hatching, and therefore would be detect possible delays of the dimension and variability we observe here. Rather, we have focused on intestinal cell endomitosis, a developmental event that is relevant at around the same time as seam cell divisions. By performing time lapse imaging, we have found that this event is not impacted by the *daf-2* mutation.

We have included this data in Fig. S3 and added the following text:

-Changes to manuscript:
"We then analyzed another cellular process that takes place around the end of the L1 stage, endomitosis of intestinal cells. During endomitosis, cell enter the M phase of the cell cycle but do not undergo cytokinesis. Endomitosis occurs during L1 in 14 of the 20 intestinal cells, produce binucleated cells (Hedgecock and White, 1985). The mutation *daf-2* does not delay the timing of this event relative to ecdysis (Fig. S3H), suggesting that this mutation specifically impact the timing of some cellular events".

2. Along the same lines, the claim that L2lag coincides with a pause in oscillatory gene expression requires more evidence and better presentation of available evidence. From the present analysis in Fig. 1H-J, it seems difficult to exclude an alternative scenario where the signal merely scales with a longer L2 in daf-2 mutants (i.e., taking longer to reach the peak and decline again, rather than exhibiting a lag/pause before increasing at normal pace). The authors must show the dynamics for the entire L2/oscillation period (i.e. going up an down) both in experimental time and scaled to the duration of each larva's L2 stage. RECOMMENDED: Their point would also be greatly strengthened by inclusion of another reporter, ideally expressed in another phase, to test whether the observation applies to a larger fraction of oscillating genes.

The reviewer is right that our previous analysis did not allow to discard a global scaling of the signal during the long L2 stage of *daf-2* mutant.

The original data we acquired did not include the complete larval stage (Ecdysis 1 to ecdysis 2) for all worms, making impossible such analysis. Furthermore, at the time we chose F11E6.3 because of the timing of signal increase corresponds to that of the beginning of intermolts, according to previous literature (https://pubmed.ncbi.nlm.nih.gov/32687264/ , Fig. S4). However, reviewer 1 raised the point that the F11E6.3 reporter is unusual in that its expression does not peak in L1.

To overcome these issues, we have now repeated these experiments using the reporter dpy-9p::GFP (HW2526) in a wild-type and *daf-2* backgrounds. In this occasion, we have acquired images from the end of L1 to the beginning of L3, therefore including the complete L2 stage. This way, we could rescale the dpy-9p::GFP signal to the duration of the L2 stage (from ecdysis 1 to ecdysis 2) *in each individual larvae*. This analysis shows that the increase in dpy-9p::GFP is delayed in *daf-2* mutant, even after rescaling to the duration of the stage.

The results from these experiments are shown in Fig. 1I-J.
- Changes to manuscript:
"We performed time-lapse microscopy of single larvae contained in 275x275x30 mm hydrogel microchambers [16] to monitor the expression of *dpy-9* in the wild type and *daf-2* mutants during the complete L2 stage. On average, the increase of *dpy-9* signal was delayed in the *daf-2* mutant (Fig. S1F). However, as the duration of the L2 stage is different between the wild type and the *daf-2* mutant, we plotted the data rescaled to the duration of each individual larva. Even after rescaling, we found that the signal increased later in *daf-2* mutants (Fig. 1J). This data supports the existence of delay in the expression of oscillatory gene expression at the beginning of L2 in *daf-2* mutants."

Panel H requires a better description - what are the individual dots? Can trajectories be discerned at the level of individual animals? It would be important to show some representative trajectories to understand the technical noise in the data and thus the quality of the fit. Why does the signal plateau - should the fit not involve an oscillatory/sinusoidal curve? In I), what are the dashed lines? We assume CI or a similar measure, but if so, it appears surprising that it would be so narrow given the variability of L2Lag - please comment. Is the left panel in J only a schematic or is the time scale on the x-axis missing?

Panel H and the type of analysis applied at that time are not included in the current version of the manuscript.

3. ESSENTIAL: The fact that the lag appears to occur predominantly in L2 immediately suggests a connection to L2d/dauer. What happens in animals committed to dauer in different ways, and/or with other daf-2 mutant alleles; is there a correlation to the penetrance of Daf-c phenotype and/or larval stage extension? Is there something special about the e1370 allele used?

This is a very relevant question that we have also asked during our work. In the new version of the manuscript, we have included the analysis of L2lag in *daf-2* mutants cultured at 22 ºC, a condition that leads to dauer formation. Indeed, we can observe cessation of developmental progression after the second molt in the luminometer. In this condition, L2lag does not increase compared to the levels at 22 ºC (Fig. 6D). We have also performed time-lapse experiments at 22.5 ºC, observing that desynchrony is like that found at 20 ºC (Fig. 6F).

This information provided in the current version discards a correlation between L2lag and the Daf-c phenotype, and we have detailed that in the text.

- Change to manuscript:

"Additionally, culture of *daf-2(e1370)* mutants at 22 ºC leads to dauer formation, an alternative state to L3 stage. The similar levels of L2lag in the *daf-2* mutant at 20 ºC and 22 ºC, as well as the similar levels of seam cell desynchrony, confirm that L2lag does not correlate with dauer formation."

Furthermore, we have additional information on the L2lag phenotypes of different *daf-2* alleles that are not included in this version of the paper.

https://www.biorxiv.org/content/10.1101/2024.03.31.587169v1.full.pdf

Our analysis showed that Class I alleles that form dauer at 22 ºC as *e1368*, *m577* and *m596* do not increase L2lag compared to the wild type. Other Class II alleles, as *daf-2*, do increase L2lag. While this information is useful and we aim to publish it together with other features of the regulation of L2lag, we think they do not add up to the current body of work presented here. Instead, we would rather keep them apart to improve clarity of the present findings.

4. Fig. 2: According to the authors' model, daf-2-dependent insulin signaling can be either rescued by mutating the effector of the pathway, DAF-16 or the upstream phosphatase DAF-18. This implies a linear pathway where daf-16 mutation should recapitulate daf-18, but this is not the case - daf-18 is a more potent suppressor of L2lag, even in wild-type animals, pointing to a DAF-16-independent function. Moreover, in Fig. 2E, it appears that L2Lag behaves differently in wild type (fully suppressed by daf-18) and daf-2 (partially suppressed). Can the authors explain these observations?

The reviewer is right that DAF-18 acts in a linear pathway where DAF-16 is the downstream effector, but this is not its only role. DAF-18 also has other functions that are independent of DAF-16 (PMID: 22916028, PMID: 26552888, PMID: 40199585, PMID: 16407400).

As the reviewer points out, for L2lag, the *daf-18* mutation exerts a stronger suppression than the *daf-16* mutation. This suggests that DAF-18 impacts L2lag through a pathway that is independent of DAF-16.

Also, L2Lag seems to be fully suppressed by the *daf-18* mutation in the wild type but not in *daf-2*. As observed in Fig. 2E, the *daf-2* mutation increases L2lag in all genetics backgrounds, suggesting that DAF-2 signals through additional pathways that impact L2lag.

We have summarized all this information in the following sentence, noting both the different levels of suppression and the different impact of the mutations in the wt vs. *daf-2* backgrounds.

- Changes to manuscript:

"For the regulation of L2lag, the *daf-18* mutant reduces the duration measured in the wild-type background [*daf-2 (+)*] (wild type vs. *daf-18* p-value=0.0018) and the *daf-2* mutant background [*daf-*

2 (-)] (Fig. 2E). In the *daf-2* mutant background, suppression by *daf-18* is stronger than that by *daf-16* (Fig. 2E). Despite this suppression, the *daf-2* mutation increases the duration of L2lag in all backgrounds (Complete statistics in File S1), even in the *daf-16;daf-18* mutant (*t-test* p=0.002) (Fig. 2E), which suggests that DAF-2 also signals to regulate L2lag independently of DAF-18 and DAF-16."

5. ESSENTIAL: Fig. 3B: The authors' claim that lin-28 RNAi does not alter the "L2lag" is interesting, but have the authors confirmed that their RNAi treatment is effective? For instance, have they verified that they see some of the previously described heterochronic mutations (ideally the skipping of the symmetric seam cell division that is critical to their interpretation)? Is the fraction of wild type animal undergoing L2lag reduced as well?)? Given that wt and daf-2 appear to behave differently with regard to L2lag (duration, penetrance, daf-18-dependence), it would seem valuable to repeat these experiments (lin14 and lin-28 RNAi) on daf-2 mutant animals.

During our experiments, we could confirm the effectiveness of the RNAi treatments as skipping of the stage specific divisions leads to the absence of that stage. This is exactly what we can measure in the luminometry assay at the level of molting. Before performing the experiments, we first tested the RNAi treatment by analyzing the number of molts. We confirmed that the treatments were efficient when we started the RNAi treatment in L4 animals of the previous generation to the one tested. In this case, larvae presented only three molts, as expected from skipping one larval stage. We then used those conditions for the experiments and confirmed that treated larvae presented only 3 larval stages.

We have included the analysis of L2lag of *daf-2* mutants upon treatment with *lin-14* and *lin-28* RNAi. In fact, the *daf-2* was included in the same experiments we performed with the wild type. Since the results are similar to that of the wild type, we did not include the analysis initially to keep the message as simple as possible. These results are now displayed in Fig. 3B and we have modified the relevant sentence to say:

Modification to the manuscript:
"we observed that *lin-14* RNAi led to a significant reduction compared to the control RNAi, in both the wild type and *daf-2".*

6. Fig 3C, D: We do not quite understand the rationale or interpretation of the lin-42 experiments. Is it clear that the results reflect a heterochronic and not a molting clock function of lin-42 (e.g, http://dx.doi.org/10.1016/j.cub.2011.10.054, https://doi.org/10.1038/s44318-025-00585-z)?

The reviewer is right that our experiments do not allow to discern between these two functions of lin-42. In the original manuscript, we had reflected this dual role of LIN-42 with the sentence:
"However, unlike *lin-14* and *lin-28*, *lin-42* is expressed periodically during larval molts and its function impacts all larval stages regulating the reiterative process of molting [22, 23]"
with the cited references been:

22. Jeon M, Gardner HF, MIller EA, Deshler J, Rougvie AE. Similarity of the *C. elegans* Developmental Timing Protein LIN-42 to Circadian Rhythm Proteins. Science. 1999;286: 1141–1146. doi:10.1126/science.286.5442.1141

23. Monsalve GC, Van Buskirk C, Frand AR. LIN-42/PERIOD Controls Cyclical and Developmental Progression of *C. elegans* Molts. Current Biology. Elsevier Ltd; 2011;21: 2033–2045. doi:10.1016/j.cub.2011.10.054

We have now included the recent publication mentioned by the reviewer:

Spangler et al., A conserved chronobiological complex times *C. elegans* development. EMBO J. 2025 44(22):6368–6396. doi: 10.1038/s44318-025-00585-z

Indeed, the results were difficult to interpret at that point in the manuscript. This is why we only concluded:
"In summary, our results show that the duration of L2lag is impacted by genes of the heterochronic pathways that control the timing of seam cell divisions *and molting*."

Subsequent information that the L2lag correlates with desynchrony between divisions and ecdysis provide a possible interpretation that we cited later in the text:

"Interestingly, this also explains our findings on how *lin-42(ok2385)* mutation impacts L2lag. Previous research showed that the *lin-42(ok2385)* increased the duration of the L1 stage but showed a timing of seam cell divisions similar to the wild-type, resulting in advanced cell divisions relative to ecdysis [22,23], which is consistent with the reduced L2lag we find in this mutant (Fig. 3D)."

To further clarify the point raised by the reviewer, we have added the sentence:
"This suggests that the effect of LIN-42 on L2lag is likely related to its molting clock function (Spangler et al.)"

7. ESSENTIAL: Fig. 4: It appears that a control is missing here; daf-18 single mutant. Without it, we find it difficult to interpret the apparent acceleration of div1 and 2 in the double mutant vs. wild type, as also pointed out in ll. 216-217.

We thank the reviewer for pointing out to this issue. The sentence in lines 216-217 was:
"The *daf-18* mutation accelerated all the events, even when compared to the wild type (Fig. 4B, S2A)."

Here, we meant that *daf-18* accelerated seam cell divisions (of the *daf-2* mutant), even compared to the wild type.

We have corrected that in the current version of the manuscript:

"However, in the *daf-2* background, the *daf-18* mutation accelerated divisions so that in *daf-2;daf-18* mutant most divisions occur before ecdysis (Fig. 4B,D)."

Nevertheless, we have performed new time-lapse experiments with the seam cell reporter strain in the wt, *daf-18* and *daf-2;daf-18* backgrounds to assess this difference. In these experiments, the *daf-18* mutant shows similar cell divisions timing as the wild type and *daf-2;daf-18*.

These results are presented in Fig. S3A of the revised version.
“The *daf-18* mutant showed a level of desynchrony similar to that of the wild type (Fig. S3A).

8. Line 208-215: The authors claim that div1 occurs at a similar time relative to hatching, while div 2 is also delayed in the daf-2 mutant, but to a larger extent than ecdysis 1. However, ecd1 is scored from hatch, while div2 from div 1 - which occurs ~5h after hatch, making for a substantially smaller denominator. Since the point that ecd1 and div2 are differentially affected is made more convincingly by Fig. 4B,D, we suggest . and the related text; if the authors prefer to keep it, we would request to reference both events to the same landmark; either hatch or div1.

We agree with the reviewer that this point is already made in Fig. 4B, D. As suggested, we have removed the two panels from the supplementary figure and the related text.

9. Ll. 220 - 222: We do not understand where/how Fig. 4E (and G) shows a correlation of the levels of desynchrony and the duration of L2lag. Where is desynchrony quantified and how? Does this refer to 4D and F respectively? If so and we eyeball the effects, it appears that div2 in Fig. 4D differs by ~2h between mutant and wt, but the lag increases by only 30 min in 4E - does this not contradict the authors' model that seam cell division needs to be completed to initiate bona fide l2? The same points apply to Fig. 5H, and contrary to the phrasing in ll. 286-287, we find it surprising how modestly (if marginally statistically significant) HU treatment extends L2lag although it delay seam cell division substantially.

We apologize for the lack of clarity here. In this sentence we refer to the correlation between the delay of seam cell division relative to ecdysis (div2-ecd1, shown in Fig. 4D) and the duration of L2lag for the same genotypes (former panel Fig. 4E). We have clarified this point in the text and also plotted the correlation analysis as Fig 4E, instead of showing L2lag values again. We have also made the text consistent with the information provided for *daf-2;daf-16* (relative to the next point raised by the reviewer).

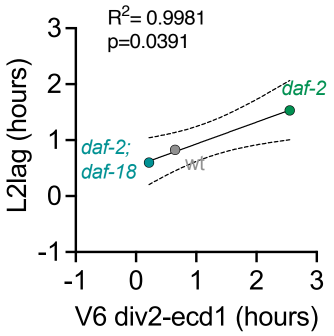

- Changes to the manuscript: "The average timing of the last divisions (V6 lineage) in the wild type occurred at 0.65 hours after ecdysis. For the *daf-2* mutant, this event happened 2.55 hours after ecdysis (*t*-test; p<0.0001) and for the *daf-2;daf-18* mutant at 0.22 hours after ecdysis (*t*-test; p=0.04). These results show that reduced insulin signaling impacts the molting and cell divisions differently, leading to a desynchronization between them. This desynchronization occurs around the transition from L1 to L2, the moment when L2lag takes place. To understand whether seam cell delay (relative to ecdysis) could be related to the L2lag phenotype, we compared their durations across genotypes, observing a statistically significant correlation (Fig. 4E)."

While L2lag does not equal seam cell division timing (relative to ecdysis) in absolute values, there is a positive correlation among these two variables when comparing the different genotypes. Indeed, from this correlation we can´t conclude that model that seam cell division needs to be completed to initiate bona fide l2. However, it is important to recall that this are independent experiments and there are factors that might have marginal effects on absolute timing of the processes. For example, illumination in the GFP excitation range, different reporters…

For these reasons, we performed the experiments in Fig. 5A-B, which confirm that larvae in L2lag show delayed divisions. This result connects the two features in individual larvae but does not show that divisions need to be completed to exit L2lag. Rather, seam cell divisions can be used as a marker or process or processes that are delayed in these larvae. We have now made this interpretation more explicitly in the manuscript.

"This result suggests that delayed developmental events, evidenced by seam cell divisions, lead to entry into L2lag."

"Taken together, our results support the idea that the initiation of larval stages can be delayed when events of the previous stage did not complete."

Regarding HU, we have also calculated the correlation between L2lag and seam cell division timing, including the conditions that reduce desynchrony (HT115 feeding in wt and *daf-2*). As before, while the two values are nor equal, there is a significant correlation between the two variables in all conditions. We have included this analysis in Fig. 5I.

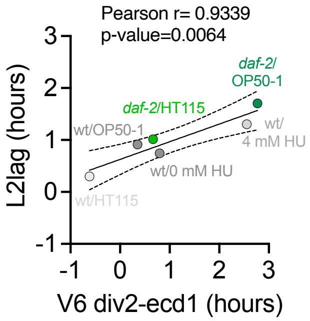

This analysis reinforces the correlation between cell division timing (relative to ecdysis) and L2lag, and shows that modulating the former impacts the timing of the latter.

10. Along the same lines, the authors state (l.237) that in the V6 division of the daf-2; daf-16 double mutant occurs after ecdysis in 56% of the animals, and conclude by saying that the result is in line with a partial rescue of the L2lag phenotype. However, it seems to us the div2 relative to ecd1 shown in Fig S2D is similar to the one shown for daf-2; daf-18 in Fig 4D, and less than what wt shows in that figure. Please state those fractions, explain whether or not there is a significant difference and whether this is then consistent with the conclusion in the following sentence. We also suggest replotting the wt from Fig. 4 for reference with the mutants in S2D.

In the current version of the manuscript, we show new data that includes the wild-type strain in Fig. S3F,G, to allow comparison of the *daf-2;daf-16* with this control. This experiments already included this control, that we didn´t show at the time to focus on the effect on the daf-2 background. Instead of replotting the wild type from a different set of experiments, this way we can properly compare the timing of V6 division in the wild type and the *daf-2;daf-16* mutant. Unlike *daf-2;daf-18*, the double mutant *daf-2; daf-16* shows a later timing of division that the wild type. We have provided these details in the text but, as the differences between the wild type and *daf-2;daf-16* are not statistically significant, we have removed the argument on the partial rescue.

"However, the acceleration from the *daf-16* mutation was not as that from *daf-18*, as the average timing of the last divisions (V6 lineage) of the *daf-2;daf-16* (0.35 hours) was close to that of the wild type (0.12 hours, t-test p=0.3)."

11. Fig. S2C, ll. 233-234: daf-2 mutation appears to mostly cause a reduction in DAF-16::GFP, not so much of a nuclear translocation. This seems unexpected, also by the authors' own preceding description of the pathway. Please explain.

The reviewer is correct that the pictures seem to show a reduction in *daf-16* expression. However, quantification of total *daf-16* would require additional analysis that does not seem necessary at the moment, as its nuclear localization that is required for the function as TF.

We present these pictures only to show that DAF-16 can be nuclear at certain points *during development on food*, especially in *daf-2* mutants.

12. Fig. 5A: This is a very nice experiment, but it lacks some technical description to fully understand it and its limitations: Is the assays stopped multiple times to extract individual worms or are many worms extracted at once? How long does it take to extract larvae, mount and watch, and were all animals manipulated and tested in parallel and from the same plate or in separate experiments etc.?

We have completed the information at the Methods section.
- Changes to manuscript:

"After approximately 20 hours of initiating, we monitored the larval developmental progress continuously. At a single timepoint, we collected larvae that were in the different categories to be analyzed. During each replicate, we extracted only 4-5 larvae from the complete 96-well plate to maintain all the processing time of the samples lower than 30 min. We collected animals that at the time of collection were within 0.5-1 hour from the previous transition, that is, from the end of M1 for animals in the categories "L2lag" and "I2", and from the end of L2lag for the larvae in the category "I2 after L2lag". Individual worms were isolated and washed from food M9. Finally, worms were immobilized using 10 mM Levamisol diluted in M9 and placed in agarose pads. Larvae with seam cell divisions were identified using a Leica scope M205 FCA and RFP excitation/emission filters."

13. Fig. 5B, ll.259-267: The experiment lacks proper description - what genotype and how many animals were assayed? From discussion (ll. 352-354) we infer daf-2, but it is not clear. Nor can we understand the legend, please rephrase. What do the different lines represent?

We apologize for the brief legend here. We have now clarified this as follows:

(B) Average bioluminescence relative to ecdysis (time=0), for larva that show divisions before (14 wt larvae) or after (18 *daf-2* and 2 wt larvae) ecdysis 1. The signal from each larva is normalized to the bioluminescence level at ecdysis (bioluminescence=1 at time=0). Continuous lines represent the average bioluminescence value. Dashed lines represent s.e.m.

Animals with divisions not yet completed at ecdysis appear to have reduced luminescence signal, as predicted by the authors' model - but the drop appears moderate. Please reanalyze the data and take the 20% or 50% of animals with lowest luc expression at ecdysis and a) plot their luminescence vs. the other animals' for comparison and b) quantify fraction with undivided seam cells for each group.

Indeed, the reduction in signal is less pronounced that the one we observe in the luminometer. The issue here is the difficulty to quantify the bioluminescence signal in the microscopy setup, as explained before (response to reviewer 1, point 1). For this reason, we have now shown all the individual plots of the animals averaged in this figure as Fig. S4. The measurement is noisy for the technical reason already detailed above, but most of the individual larvae in the different groups show the trend that is later observed in the grouped data.

Nevertheless, the combination of animals with different duration of L2lag as measure in the luminometer results in a drop in luminescence signal that is similar to that we find in the imaging experiments.

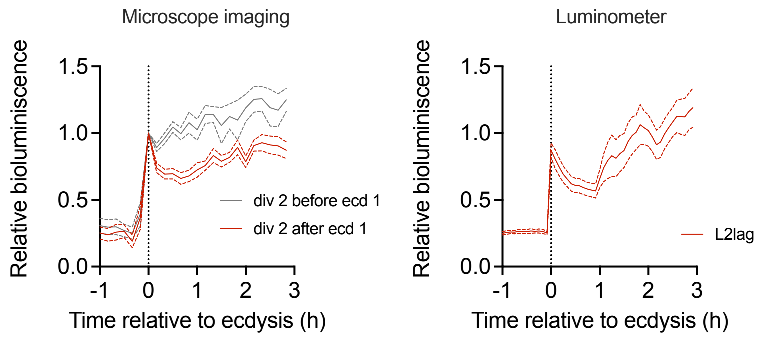

14. We also infer from Fig 5B that animals with a lag phase also appear to have an extended M1 molt - is this correct? This is not obvious from Fig 1C and D - can the authors please comment? If this is a general feature, it would be helpful to mention it with introduction of Fig. 1, if not, the difference needs to be explained.

In the plot this stemmed mostly from the abundance of *daf-2*. While *daf-2* mutants have a slightly longer M1 than the wild type, the duration of M1 does not correlate with that of L2lag neither in wt nor in *daf-2* mutants (Fig. S1D,E).
To avoid this confusion, we have plotted the signal from the last hour of M1.

15. The authors show that HT115 as a food source accelerates seam cell division, but it seems that V6 cells still divide after ecdysis in daf-2 on this food - with point 9 and 10 above, this would seem to argue against the simple "seam cell division check point" model?! Please also reference literature showing accelerated seam cell division (or if a new discovery of the present study, state so.).

As explained in the reply to point 9 and 10, the data regarding HT115 also fits the correlation between L2lag and seam cell divisions, even when the absolute times are not the same.

We have included a reference for the acceleration of development by HT115 and also stated that, additionally, we unveiled a further acceleration of the divisions that led to a situation where most divisions occur before ecdysis.

- Changes to manuscript:

“HT115 accelerate the timing of postembryonic development (PMID: 33159120), but its role on seam cell division has not been assessed. While feeding with HT115 might impact additional processes, we could establish that HT115 significantly advanced seam cells divisions compared to OP50-1, in both the wild type and *daf-2(e1370)* (Fig. 5C), without impacting the timing of ecdysis (Fig. S5A). This led to a situation where most divisions occur before ecdysis (Fig. 5D).”

16. Fig 5I: The authors aim to show that L2lag is an independent mechanism that does not scale with larval stage duration by evaluating L2lag on the population level for different growth temperatures. OPTIONAL: They could strengthen this argument by evaluating it for single animals,

ideally correlating the L2lag with timing in seam cell division (optional but highly interesting). OPTIONAL: Moreover, it would be useful to see the effect of temperature in daf-2 mutants, where the two timers are desynchronized (ditto).

We have performed time-lapse experiments at 22.5 ºC to measure the timing of seam cell divisions relative to ecdysis. In these experiments we found no differences in timing of divisions compared to that found at 20 ºC. As suggested by the reviewer, these results support the idea that L2lag does not scale with the duration of the larval stage but correlates with the level of seam cell desynchrony. Additionally, we have analyzed the effect of temperatures in the *daf-2* background for L2lag, and performed time-lapse for 22 ºC for this mutant. As in the wild type, L2lag does not present significant differences between temperatures.

We have incorporated these results to Fig. 6A-F.

17. The discussion needs to be improved. It seems unnecessary to have a long paragraph on the virtues of the luminescence assay (first paragraph of Discussion). More importantly, two pages of discussion on the "differential impact of IIS on developmental events" seem particularly excessive when the entire conclusion relies on only one perturbation (daf-2(e1370)) of IIS signaling. We find the last paragraph (ll. 369 - 378) particularly confusing as it appears to imply that insulin signaling is an integral component of L2lag generation or determination. This seems at odds with the remainder of the manuscript arguing that perturbed insulin signaling affects seam cell division more strongly than molting, necessitating the lag phase for realignment - i.e., IIS perturbation is a tool to uncover L2lag, not a mechanism controlling it.

We have worked on the discussion along the lines proposed by the reviewer.

18. To make a general claim of modularity of the system, we think stronger/additional data would be needed - going beyond L2 (where most of the current data was collected) and focusing on events that go beyond seam cell division and ecdysis. If these data are not available, we suggest strongly toning down, or omitting entirely, this part.

We have toned down the references to modularity and restricted it to:
"Such compensation confines perturbations to a single stage, resembling developmental modularity, which enhances evolvability by allowing independent optimization of stage-specific processes (reviewed[35])."

Minor points/typos/presentation:
1. Some bar charts seem to have a lot of overlapping data points, particularly around 0 (e.g. Fig. 1E) that end up being highly overplotted - perhaps a violin plot offers better representation of the data structure.

We thank the reviewer for the feedback, but we have tested several ways to represent this data over the years seems to be the clearer and the most transparent way to show the values of each animal. While the high number of larvae leads to overlapping of the individual measurements, the average values showed in black throughout the manuscript allow a clear visualization of the general results.

Furthermore, this type of representation better adheres to the PLOS Biology recommendations for data presentation.

2. Line 41-42: consider rephrasing, currently sounds as if intermolt is part of molt.

We have changed this sentence to:
"Additionally, across the four larval stages (L1–L4), development proceeds through a repeated cycle in which each stage includes an intermolt period (I1–I4) and a subsequent molt (M1–M4)".

3. Line 72-74: "intermolt" in its literal meaning is the time between two molts; hence, it is a bit unclear what is meant by "delaying the initiation of the following intermolt"

We have modified this sentence to:

"Our results suggest a model in which delayed cell divisions can be compensated by delaying the initiation of the following stage, so that desynchrony does not amplify over the process of development."

4. Line 84: "bocks" => "blocks"
Corrected

5. Fig. 1G: Is this needed? The effect is not striking and there does not really seem to be a conclusion by the authors.

We have removed this panel and the related text.

Also the Table S1 referenced in the legend should probably be S2 and we could not find a File S1 (referenced also ll. 170-173).
Corrected

6. L. 120: "Lags occurs" => "lag occurs, and later "larva stages" => "larval stages". Moreover, please recheck references for this sentence. References 16 and 18 are written in round brackets and it appears to us ref. 24 should be the ref. to cite here.

Corrected

7. L. 125 and elsewhere: "concealed" => "contained"
Corrected

8. Fig. 2A is not very accessible. A "split" figure where one panel shows the situation with active, one with inactive DAF-2 signalling would probably help. What are the grey blobs? Either label and explain all components or, preferred, simplify. Ll.154-156 refers to the interaction between DAF-18 and AGE-1, so AGE-1 should probably be labeled in the figure.

We have changed this panel to a split figure, removed the proteins not mentioned in the text and include AGE-1, which is mentioned.

9. L. 144: "...phenotypes observed in daf-2 mutants as increased duration of the increased duration

of the L2 stage, are absent in the double mutant daf-2(e1370);daf-16(mu86)". The authors showed in Fig 2C that the L2lag is only partially rescued, so the overall larval stage should still be longer?!

From the references mentioned, the one that concerns the duration of development is PMID: 20944013. Here, duration of the stages was calculated by inferring the molts from the fraction of larvae not pumping within a synchronized population. As a reference, in that article, the duration estimated for *daf-2* is 20 hours, and for the *daf-2;daf-16* is 10 hours, with no decimal points nor errors calculated.

10. L. 146: "... show a duration of the second intermolt... (Fig 2B)". intermolt => Bona fide intermolt
Corrected

11. Ll. 154-156: please state that DAF-18 is PTEN in worms
Included in the text: "Looking for additional regulators of the process, we focused on DAF-18, the *C. elegans* homologue of the tumor suppressor PTEN..."

12. L. 205: "Using strains with the reporter matIs38..." - please indicate what this transgene exactly "reports" on; it appears to be a rather complex transgene array.

We chose this reporter because it includes an mCherry marker for seam cells besides the GFP marker. We use the signal from mCherry to monitor the division of the seam cells in the strain that combines this with the bioluminescence reporter, which also contains GFP. *Pscm::CYB-1 DB-mCherry::unc-54 3' UTR* reports for cell cycle progression, been present in S phase and G2, but not at the end of mitosis and G1. In our case, we use it only used for visualization of the seam cells, without analyzing the intensity of the signal.
Finally, *Pmyo-2*::GFP is in the construct as a marker of the transgene, as the expression form *Pscm* is not equally visible throughout the lifetime of the animal.

Importantly, we have tested:

-That the duration of development is not affected by *matIs38* by comparing the double reporter *matIs38;sevIs1* with *sevIs1* alone.

- The delay between ecdysis and seam cell divisions in a reporter with only a GFP (Pscm:GFP), for the wt and *daf-2* backgrounds, and the results are the same as with the one used here.

13. Fig 4A: micrographs are too small
We have increased their size. The function of these is only to show the two features that are analyzed, ecdysis from the cuticle and seam cell divisions.

14. L. 225: "pronounced that" => "pronounced than"
Corrected

15. L. 263: "different" => "difference" and later "... between animals had already..." => "between animals that had already..."
Corrected

16. Ll. 328-331: "a brake ... at the level of molting" - since molting appears to occur at the normal time and for the normal duration, it is unclear that there is a specific brake on molting (rather than on intermolt).
Here, when we wrote "at the level of molting" we referred to the complete process, including molts and intermolt. The reviewer is right that this could causes some confusion, so we have changed the sentence accordingly:

"...developmental progression can pause at the beginning of intermolts,"

17. L. 425: call-out should presumably be to Table S1
Corrected

18. L. 461: "sticked" => "stuck"
Corrected

19. Ll. 466-467: parenthesis make the sentence hard to read
Rephrased as follows:

"After eggs were transferred, we collected some OP50-1 from NGM plates using the eyelash and added it to wells as food source. The only exception is for Fig. 5C and S5A, that we used OP50-1 or HT115."

20. Ll. 495-509: Luminescence recording: we are unsure how this was done. If signal was integrated over 1 min, the worm would be expected to have moved substantially, so how is the ROI then defined from the transmitted light image?

As the reviewer acknowledges, the worms move during that minute, and therefore we have to draw a ROI over the entire (microchamber) well.

To clarify this, we have modified this part of the text in the methods section.
- Changes to manuscript: (pending)

"For bioluminescence, we integrated the signal form 1-min exposure. To obtain the average bioluminescence signal we use the ImageJ software. *As larvae move over the 1-min exposure*, we draw a region of interest (ROI) that covers the entire microchamber well to integrate the signal from the complete acquisition time, then obtaining the average bioluminescence value per pixel."

Rev. 3:

In this manuscript, the authors establish that developmental programs in C. elegans, such as seam cell division and ecdysis, are regulated independently and a loss of synchrony between these processes can lead to a subsequent delay in the molting program that allows resynchronization of development. This prevents a knock-on effect whereby all subsequent developmental larval stages could be adversely affected by earlier delays. This suggests a degree of developmental flexibility and the existence of mechanisms that allow coordination of developmental timing across different tissues and processes. These findings have important implications for understanding the regulation of development and are therefore likely to be appreciated both in the C. elegans field and beyond.

The authors use several innovative approaches to characterize developmental timing in individual animals. The analysis of cell division timing relative to molts is detailed and comprehensive. The use of the luciferase reporter strain is fundamental to the identification of the lag periods and is a novel application of this strain. This reporter has previously been validated by the authors as a marker of larval growth and the timing of molts. During the molts, luminescence is diminished due to the inability to ingest the luciferin substrate. In the current study, the authors determine that many animals have a delay in the restoration of the luminescent signal after ecdysis, despite completion of the molt, which they define as a lag phase. This lag phase occurs in a significant fraction of wild-type animals but is exacerbated in mutants that affect the insulin signaling pathway, as well as being influenced by nutritional status, with considerable inter-individual variability in lag duration.

My main question is whether this reporter is providing a readout of a fundamental biological process that underpins the observed lag period or whether we are observing an artificial, construct-specific phenomenon that happens to correlate with seam cell division and ecdysis? With respect to this question, is it possible to provide an orthogonal measure, independent of the luciferase reporter, that demonstrates the existence of the lag phase? For example, does the L2lag cause a delay in the resumption of growth at the level of the whole body?

In the new version of the manuscript, we have worked on further characterization of the lags, showing, among other things, that during L2lag larvae move less than during I2 but more that during M1 (Fig. 1F) and that they keep pumping and growing between ecdysis 1 and division 2 (Fig. S3C,D).

Nevertheless, L2lag is most relevant as a marker of paused molting. This is indeed a marker that correlates with the delay in seam cell divisions. What is key is that L2lag is a period of time that is added to the (*bonafide)* intermolt, as opposed to a feature accommodated during the average duration of the regular intermolt.

We have now added new analysis to reinforce this idea (Fig. S1F), based on the duration of the L2lag and *bf*. I2. Importantly, we performed this analysis on wild-type larvae, to show how this is independent of possible *daf-2* related phenotypes as reduced feeding.

For larva that present L2lag, if we represent the duration of the complete intermolt, strictly considering the time between molts, its duration is significantly higher than that of larvae that do

not enter this stage. By subtracting the duration of the L2lags, we obtain a *bonafide* I2 intermolt duration that is statistically not different from the average I2.

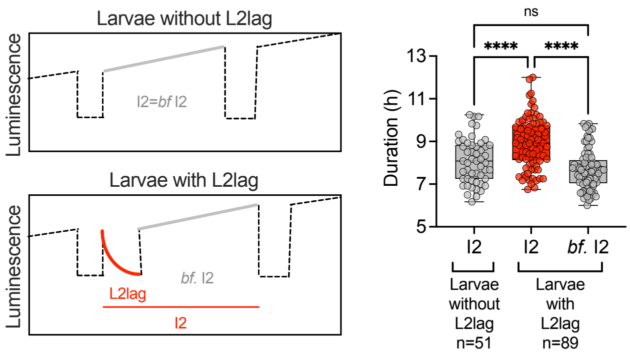

Additionally, we commonly synchronize larvae of N2 (wild type) and observe that in plates with around 1000 animals there are usually a few that look significantly delayed. We have quantified that for N2(wild type) and CB1370(*daf-2*), observing around 1-2% and 5-8% delayed animals respectively. These numbers are low compared to the levels of L2lag but in this case the visual inspection only allows to detect those with strong delays (possibly very long L2lag).

Our observations from the luminometry assay explain that these are not just "overall slow" larvae. Importantly, only a time-resolved, single-larva method as the one we apply allows observing this type of features of development. As such, this featured of development has passed unnoticed for a long time.

Finally, a nutritional checkpoint has been proposed to exist at the beginning of larval stages. When larvae are deprived of food mid larval stage, the developmental event continue up to the beginning of the next larval stage (Schindler, Baugh, & Sherwood, 2014). Furthermore, the model for a molting oscillator in *C. elegans* predicts a checkpoint at this same stage (Meeuse et al., 2020), but no further data has been gathered regarding this topic. Our data reinforces the idea of a point of developmental control at the beginning of larval stages, but it expands its biological significance, as we show that these could be used as checkpoints that allow resynchronization between developmental events.

The Psur-5::luc+::GFP transgene is an exogenous construct integrated into the C. elegans genome and the sur-5 promoter is described as constitutive. A previous study by the same authors showed that the molt does not affect luciferase protein expression in wild type worms. However, sur-5 gene expression does change during development, including during dauer formation, a process that is affected by changes in nutritional state and the daf-2 insulin signaling pathway. There also appear to be DAF-16 binding sites in the sur-5 promoter. Moreover sur-5 encodes a protein that, according to Wormbase, is predicted to enable acetoacetate-CoA ligase activity and be involved in a fatty acid metabolic process. Based on this, is it possible that sur-5 promoter activity is subject to nutritional regulation that affects luciferase expression after the molts in the various backgrounds?

With this in mind, it would be useful to determine to what extent luciferase protein levels are affected by the daf-2 mutation, DAF-16 activity or nutritional state (OP50 vs HT115). Could the same lag phase be demonstrated with luciferase driven by different constitutive promoter?

We thank the review for the comment, which stimulated us to perform two different experiments.

The reviewer is completely right that the decreased luminescence signal might be due to a decrease in the expression of the luciferase enzyme, what is easily measured by quantification of GFP (LUC::GFP fusion protein).
However, it is important to remind that the overall level of signal is not relevant to define L2lag. In order to know whether low levels of luciferase are responsible for the low signal during L2lag, more than analyzing luciferase protein levels under different genotypes or feeding conditions, it is key to measure whether these levels change during L2lag.

To investigate this issue, we have measured GFP levels at different moments, specifically M1, L2lag and I2. To collect animals at these specific moments we used the same approach as presented in figure 5A of the previous version. We followed development in the luminometer and extracted larvae from wells at the indicated moments and acquired pictures to measure the GFP signal. In this experiment, we observe similar levels of GFP, concluding that the decrease in luminescence during L2lag signal does not come from a reduction in expression of LUC::GFP. The results have been included as a further characterization of L2lag in Figure 1E, and are accompanied by the following text:

-Changes to manuscript:

"To analyze whether the reduction of the luminescence signal during the lags stems from a reduced expression of the luciferase protein under the promoter of *sur-5*, we measured GFP in animals collected during the first molt (M1), L2lag and the second intermolt (I2). The three categories showed similar levels of GFP expression, suggesting that the decrease of the signal is not a consequence of reduced luciferase expression (Fig. 1E)."

Additionally, to demonstrate that the lags also occur when luciferase is driven by different constitutive promoter, we have analyzed L2lag in strains containing *xeSi296[eft-3p::luc::gfp::unc-54 30UTR, unc-119(+)]* II. This strain holds a single copy of luc::gfp integrated by MoSCI. Furthermore, the promoter is that of *eft-3*, different from the *sur-5* promoter in the original strains. By running the two strains in parallel in both the wild-type *and daf-2(e1370)* genetic backgrounds, we observe the same duration of larval stages and L2lag. We have included this information in the new version of the manuscript as Fig. S1A.

This result been included in Fig. S1A and are accompanied by the following text:

"Furthermore, to confirm that L2lag is not construct specific, we analyzed another luciferase strain with a single-copy insertion and a different promoted driving the expression of luciferase [4]. L2lag and larval stages show the same duration in both the wild-type and *daf-2(e1370)* backgrounds (Fig. S1A)."

The identity of the biological factor(s) that underpin the lag response remains an unanswered question in this study. Although their identification may ultimately be beyond the scope of this manuscript, there are several candidates that could be assessed fairly trivially. For example, on completion of the molt, does pharyngeal pumping restart immediately and, if so, is luciferin immediately available in vivo? Is there variability in the timing of the resumption of pumping or the efficiency of pumping that limits ingestion and / or bioavailability of luciferin? If the luciferase protein level is unchanged, and luciferin is bioavailable, does that mean that ATP availability is the rate-limiting factor? If so, does this suggest that the desychrony is fundamentally linked to ATP generation and therefore associated with mitochondrial function?

We appreciate the reviewer's insightful comments regarding the biological determinants of the lags. We agree that the precise identity of the factor(s) underlying this lag remains unresolved. We propose the lags result from desynchronization of developmental events and we are always planning how to face the challenge to unveil the mechanisms that leads to the lags. In that sense, we are mostly interested understanding how the initiation of larval stages is paused upon delay of other developmental processes. The lags will be the readout for the paused state. While we would also like to understand why the bioluminescence signal is low during the lags, we think this is not trivial. Once we have discarded that the LUC expression is reduced during the lags, the other factors that contribute to the signal are indeed the substrate availability and ATP levels.

Regarding the question whether pharyngeal pumping restarts immediately after completion of the molt, we understand the reviewer refers to the larvae in L2lag. Then, within the luminometer setup, if we collected larvae immediately after completing the molt to measure its pumping rate, it would not be possible to determine whether they will subsequently enter L2lag or proceed to the intermolt. Within our time-lapse imaging system, we observe pumping and have now included this information in the text. However, to know whether pumping restart immediately after ecdysis would imply detecting the exact moment of molt completion and then continuously monitoring pharyngeal pumping at the necessary temporal resolution, what would require uninterrupted imaging for extended periods. Because molt timing varies markedly between individuals, we would have to capture the entire pre-molt interval across all animals. Furthermore, accurately detecting pumping requires imaging at a frame rate compatible with pump rates of up to ~200 pumps per minute, which substantially increases phototoxicity risk and data burden. These requirements go well beyond the scope and technical design of our current imaging assays.

Similarly, establishing whether luciferin is immediately bioavailable in vivo, as well as ATP availability are things we could face when stablishing the methods to measure development, but that don´t seem immediately addressable at the level of individual larvae, which would be required for any analysis regarding L2lag, due its high interindividual variability.

In the Discussion of the new version, we have included a sentence stating that our current assays cannot resolve the mechanisms underlaying reduced luminescence signal during the lags. We agree that dissecting these mechanisms might shed light into the generation of the lags.
