# Supplementary material for: A desynchrony mechanism between events triggers a compensatory delay during Caenorhabditis elegans development
Source: PLoS Biol. 2026 Jul 24;24(7):e3003867. doi: 10.1371/journal.pbio.3003867 (PMC13399346; doi:10.1371/journal.pbio.3003867)
Supplement: Attachment — Submitted filename: Response to reviewers_2.pdf [file pbio.3003867.s013.pdf]

Romero-Expósito et al., (PBIOLOGY-D-25-02981R2) Response to Reviewers

We thank the reviewers for their time and dedication to improve the manuscript.

Reviewers' comments
Rev. 1:
I am satisfied with the new experiments added and the authors' rebuttal successfully resolves my concerns. I have no further comments and recommend publication.

Rev. 2:
- The authors have largely addressed our criticisms, and we appreciate the effort put into the revisions. What is currently missing is a (clear) description of the new figure panels 4E and 5I. We understand that some correlation is shown, but the underlying model, and the identity of the curved lines, are unclear. We are willing to take the authors' word that it supports their claim, but this should be clarified in the published version.
The strong correlation in the values of L2lag and div2-ecd1 across genotypes and conditions supports that these two features do not vary independently. Instead, their durations are tightly linked and change proportionally. This is exceptional, as L2lag does not change proportionally to the duration other larval stages and demonstrates that variation in the duration of the L2lag could reflect changes in the timing of seam cell divisions relative to ecdysis.
At this point, this analysis only aims to prove said correlation.
Later in the manuscript, we modify the div2-ecd1 period to measure the reaction of the L2lag duration.
We have modified the description of this analysis in the manuscript to try improving clarity:
Changes to text:
"To understand whether seam cell delay (relative to ecdysis) could be related to the L2lag phenotype, we compared their durations. We plotted the L2lag duration against the V6 div2–ecd1 interval for wild type, *daf-2*, and *daf-2;daf-18* animals. We observed a strong positive linear correlation between these variables across genotypes (R^2 = 0.9981, p = 0.0391), demonstrating that variation in the duration of the L2lag could reflect changes in the timing of seam cell divisions relative to ecdysis (Fig. 4E)."

The curved, dashed lines correspond to the 95 % confidence interval, as we have now stated in the figure legend.
Changes to figure legend: "Dashed lines correspond to the 95 % confident interval."

- The statement in ll. 314 - 318 is rather opaque, can the authors rephrase it to clarify what "was not as that from daf-18" means - and whether their data support or don't support their model?
We apologize for the lack of clarity and have edited the text to solve this issue.

(Call-outs should also be corrected to Fig. S3 rather than S2, both for this entire paragraph and the subsequent one.)
Corrected

Changes to text:
"The *daf-16* mutation accelerated seam cell divisions of *daf-2(e1370)* similarly to their impact on L2lag (Fig. S3F,G). However, **the acceleration from the *daf-16* mutation was not as strong as that provoked by the *daf-18* mutation**..."

Rev. 3:
Accept.
