# Supplementary material for: A desynchrony mechanism between events triggers a compensatory delay during Caenorhabditis elegans development
Source: PLoS Biol. 2026 Jul 24;24(7):e3003867. doi: 10.1371/journal.pbio.3003867 (PMC13399346; doi:10.1371/journal.pbio.3003867)
Supplement: Peer review history

# Editor report: Decision Letter 0

**DOI:** 10.1371/journal.pbio.3003867.r001

**Authors:** Ines Alvarez-Garcia

28 Sep 2025

Dear Dr Olmedo,

Thank you for submitting your manuscript entitled "Desynchrony between events triggers a compensatory delay during C. elegans development" for consideration as a Research Article by PLOS Biology.

Your manuscript has now been evaluated by the PLOS Biology editorial staff as well as by an academic editor with relevant expertise and I am writing to let you know that we would like to send your submission out for external peer review.

However, before we can send your manuscript to reviewers, we need you to complete your submission by providing the metadata that is required for full assessment. To this end, please login to Editorial Manager where you will find the paper in the 'Submissions Needing Revisions' folder on your homepage. Please click 'Revise Submission' from the Action Links and complete all additional questions in the submission questionnaire.

Once your full submission is complete, your paper will undergo a series of checks in preparation for peer review. After your manuscript has passed the checks it will be sent out for review. To provide the metadata for your submission, please Login to Editorial Manager (https://www.editorialmanager.com/pbiology) within two working days, i.e. by Sep 30 2025 11:59PM.

If your manuscript has been previously peer-reviewed at another journal, PLOS Biology is willing to work with those reviews in order to avoid re-starting the process. Submission of the previous reviews is entirely optional and our ability to use them effectively will depend on the willingness of the previous journal to confirm the content of the reports and share the reviewer identities. Please note that we reserve the right to invite additional reviewers if we consider that additional/independent reviewers are needed, although we aim to avoid this as far as possible. In our experience, working with previous reviews does save time.

If you would like us to consider previous reviewer reports, please edit your cover letter to let us know and include the name of the journal where the work was previously considered and the manuscript ID it was given. In addition, please upload a response to the reviews as a 'Prior Peer Review' file type, which should include the reports in full and a point-by-point reply detailing how you have or plan to address the reviewers' concerns.

During the process of completing your manuscript submission, you will be invited to opt-in to posting your pre-review manuscript as a bioRxiv preprint. Visit http://journals.plos.org/plosbiology/s/preprints for full details. If you consent to posting your current manuscript as a preprint, please upload a single Preprint PDF.

Feel free to email us at plosbiology@plos.org if you have any queries relating to your submission.

Kind regards,

Ines

--

Ines Alvarez-Garcia, PhD

Senior Editor

PLOS Biology

ialvarez-garcia@plos.org

# Peer review: Decision Letter 1

**DOI:** 10.1371/journal.pbio.3003867.r002

**Authors:** Ines Alvarez-Garcia

17 Dec 2025

Dear Dr Olmedo,

Thank you for your patience while your manuscript entitled "Desynchrony between events triggers a compensatory delay during C. elegans development" was peer-reviewed at PLOS Biology. Please also accept again my sincere apologies for the delay in sending you our decision. The manuscript has now been evaluated by the PLOS Biology editors, an Academic Editor with relevant expertise, and by three independent reviewers.

The reviews are attached below. As you will see, the three reviewers find the conclusions interesting, but they also raise several concerns that would need to be addressed before we can consider the manuscript for publication. Reviewer 1 raises several concerns including the validation of the L2Lag with an alternative method to confirm its existence, checking if the animals are not eating much during the L2Lag and, if so, why, and acknowledging the limitation that the experiments performed cannot explain why daf-16 only partially suppresses the L2Lag. Reviewer 2 thinks that the L2Lag phase should be better characterised to see if it is a behavioural or a developmental phenomenon, and also that the dynamics for the entire oscillation periods should be shown. In addition, this reviewer suggests the use of a reporter expressed in another phage to test if the observation affects a bigger fraction of oscillating genes, among other issues and clarifications. Reviewer 3 mentions that it should be determined to what extend luciferase protein levels are affected by the daf-2 mutation, DAF-16 activity or nutritional state, and that the mechanisms involved in the lag response should be also explored.

In light of the reviews, we would like to invite you to revise the work to thoroughly address the reviewers' reports. Given the extent of revision needed, we cannot make a decision about publication until we have seen the revised manuscript and your response to the reviewers' comments. Your revised manuscript is likely to be sent for further evaluation by all or a subset of the reviewers.

In addition to these revisions, you will need to complete some formatting changes, which you will receive in a follow up email. A member of our team will be in touch with a set of requests shortly.

We expect to receive your revised manuscript within 3 months. Please email us (plosbiology@plos.org) if you have any questions or concerns, or would like to request an extension.

At this stage, your manuscript remains formally under active consideration at our journal; please notify us by email if you do not intend to submit a revision so that we may withdraw it.

**IMPORTANT - SUBMITTING YOUR REVISION**

Your revisions should address the specific points made by each reviewer. Please submit the following files along with your revised manuscript:

1. A 'Response to Reviewers' file - this should detail your responses to the editorial requests, present a point-by-point response to all of the reviewers' comments, and indicate the changes made to the manuscript.

*NOTE: In your point-by-point response to the reviewers, please provide the full context of each review. Do not selectively quote paragraphs or sentences to reply to. The entire set of reviewer comments should be present in full and each specific point should be responded to individually, point by point.

You should also cite any additional relevant literature that has been published since the original submission and mention any additional citations in your response.

2. In addition to a clean copy of the manuscript, please also upload a 'track-changes' version of your manuscript that specifies the edits made. This should be uploaded as a "Revised Article with Changes Highlighted" file type.

3. Resubmission Checklist

When you are ready to resubmit your revised manuscript, please refer to this resubmission checklist: https://plos.io/Biology_Checklist

To submit a revised version of your manuscript, please go to https://www.editorialmanager.com/pbiology/ and log in as an Author. Click the link labelled 'Submissions Needing Revision' where you will find your submission record.

Please make sure to read the following important policies and guidelines while preparing your revision and fulfil the editorial requests:

a) *PLOS Data Policy*

Please note that as a condition of publication PLOS' data policy (http://journals.plos.org/plosbiology/s/data-availability) requires that you make available all data used to draw the conclusions arrived at in your manuscript. If you have not already done so, you must include any data used in your manuscript either in appropriate repositories, within the body of the manuscript, or as supporting information (N.B. this includes any numerical values that were used to generate graphs, histograms etc.). Please also indicate in each figure legend where the data can be found. For an example see here: http://www.plosbiology.org/article/info%3Adoi%2F10.1371%2Fjournal.pbio.1001908#s5

b) *Published Peer Review*

Please note while forming your response, if your article is accepted, you may have the opportunity to make the peer review history publicly available. The record will include editor decision letters (with reviews) and your responses to reviewer comments. If eligible, we will contact you to opt in or out. Please see here for more details:

https://blogs.plos.org/plos/2019/05/plos-journals-now-open-for-published-peer-review/

c) *Blot and Gel Data Policy*

Please provide the original, uncropped and minimally adjusted images supporting all blot and gel results reported in an article's figures or Supporting Information files. We will require these files before a manuscript can be accepted so please prepare them now, if you have not already uploaded them. Please carefully read our guidelines for how to prepare and upload this data: https://journals.plos.org/plosbiology/s/figures#loc-blot-and-gel-reporting-requirements

d) *Protocols deposition*

To enhance the reproducibility of your results, we recommend that if applicable you deposit your laboratory protocols in protocols.io, where a protocol can be assigned its own identifier (DOI) such that it can be cited independently in the future. For instructions see: https://journals.plos.org/plosbiology/s/submission-guidelines#loc-materials-and-methods

Thank you again for your submission to our journal. We hope that our editorial process has been constructive thus far, and we welcome your feedback at any time. Please don't hesitate to contact us if you have any questions or comments.

Sincerely,

Ines

--

Ines Alvarez-Garcia, PhD

Senior Editor

PLOS Biology

ialvarez-garcia@plos.org

------------------------------------

Reviewers' comments

Rev. 1:

In this manuscript, Romero-Exposito and colleagues describe a new transient larval pause state in C. elegans post-embryonic development. This state occurs between the L1 molt and the L2 intermolt, therefore termed L2Lag. Although this pause is initially identified in daf-2 mutants that display slow development, the L2Lag state is also detectable in wild-type animals grown under normal conditions, albeit at lower rates. The authors propose that this pause allows distinct developmental events to synchronize before initializing the next larval stage. In particular, they observe that the L2Lag tends to occur when seam cell divisions are delayed relative to ecdysis. Although the underlying cause and physiology of the L2Lag could be better described, this body of work represents an interesting finding that would be of broad interest to the developmental biology community and should be suitable for publication after minor revisions.

Major comments:

1. The L2Lag is a feature of the luminometry assay. It would be extremely important to validate its existence with an alternative method. The experiments with the F11E6.3 reporter in Fig1h-j represent a good initial step towards this goal. However, it is currently not possible to assess whether the delay in F11E6.3 induction observed in some worms by fluorescence microscopy corresponds to the L2Lag detected by luminometry or not. The authors should perform dual luminometry and fluorescence microscopy in microchambers using the F11E6.3 reporter. This should be technically feasible as evidenced by Fig5b. Such cross-validation would eliminate any doubt that the L2Lag is an artifact of the luminometry assay.

2. In terms of the worm physiology, what is happening during the L2Lag that prevents luminescence from rising? The assumption would be that the worms are not eating much during the L2Lag. The authors should determine if this is indeed the case and identify its underlying cause. Is the cuticular plug still blocking the mouth during the L2Lag? Or is this a behavioral phenotype caused by low pharyngeal pumping rates? More generally, what do the larvae look like during this state? It would be nice to see a few pictures of animals in L2Lag in the supplement. Do the larvae grow volumetrically during L2Lag or not?

3. The data shown in Fig. 4 is beautiful and very convincing that there is a correlation between desynchrony of seam cell divisions relative to ecdysis and the L2Lag state. The authors then argue that the partial suppression of the L2Lag in daf-2;daf-16 double mutants compared to daf-2;daf-18 is caused by the residual delay of V6 cell division in the daf-16 background in Fig.S2d. This argument appears in lines 236-239. However, both wild-type animals and daf-2;daf-18 mutants show similar levels of V6 division delay as daf-2;daf-16 mutants. Hence, this cannot possibly be the cause of the partial suppression of L2Lag by daf-16. The authors should remove this argument from the manuscript and simply acknowledge they cannot explain why daf-16 only partially suppresses the L2Lag.

4. The authors should also acknowledge that the perturbations they use to accelerate or delay seam cell divisions (HT115, HU) are non-specific and could be impacting many other aspects of animal development beyond their intended target. The language regarding causality should therefore be toned down in light of the limitations of the perturbations used.

Minor comments:

1. The authors should justify their choice of the F11E6.3 reporter for molting oscillations. This oscillator is unusual in that it only peaks in the L2-L4 stages, but not in L1. Why not use other reporters that peak in all larval stages, therefore allowing better detection of pauses in oscillatory dynamics, like dpy-9 or qua-1?

2. For the F11E6.3, why is a plateau observed after induction instead of the expected oscillatory profile?

3. The section describing the epistasis analysis between daf-2, daf-16, and daf-18 is written in a confusing way. Especially the sentence in lines 166-168. The authors should clarify what they mean using simpler language.

4. The variability of the L2Lag duration seems to decrease in some genetic backgrounds (e.g. daf-18). It would be nice for the authors to perform CV analysis as in Fig. 1f for the other conditions tested.

5. The lin-42(ok2385) mutant seemingly shows the strongest suppression of L2Lag among all the genotypes tested. If possible, it would be interesting to know if daf-2 mutation can still lengthen the L2Lag in lin-42(ok2385) background? This is an optional experiment.

6. In Fig. 5a, it is unclear when exactly the animals were collected for seam cell division analysis. Can the authors please add an arrow noting the time of collection?

7. For Fig.5b, tracks from individual animals should be separately shown in the supplement (i.e. one graph per animal) with the timing of seam cell divisions overlayed on the luminescence profile. This will allow readers to assess the correlation on a per-animal basis.

8. Fig. S3a shows relative timing of ecdysis - relative to what?

9. Fig. S3e, there does seem to be a trend towards delayed ecdysis with HU treatment. What is the p-value? Are there outliers driving the lack of significant difference?

Rev. 2:

In their study, Romero-Exposito and colleagues investigate mechanisms affecting the synchrony between different developmental events in C. elegans larvae, specifically focusing on the divisions of hypodermal seam cells and molting (ecdysis). Using a previously developed luciferase-based assay, where lethargus/molt can be distinguished from the animal's growth/intermolt phase by the absence/presence of luminescent signal, they discover that some animals exhibit an in-between pattern, with a period of intermediate luminescence levels following ecdysis. This pattern, termed L2lag, is particularly pronounced and penetrant when examining the beginning of L2, and in daf-2 mutant animals, which are impaired in insulin/IGF1 signaling. To elucidate the L2lag mechanism, the authors explored the contribution of different components of the insulin/IGF1 signaling pathway, namely the transcription factor DAF-16 and the phosphatase DAF-18. By combining luminescence analysis, imaging, genetics and chemical perturbations, Romero-Exposito et al. develop a model in which cell division and ecdysis are controlled by distinct timers, responding differently to perturbations such as daf-2 mutation. To resynchronize them after perturbation, an undefined mechanism comes into play and initiates the L2lag, pausing the molting timer, thus preventing the amplification of the desynchrony over the course of the development.

The idea of distinct timers that achieve synchronization by putting one "on hold" is appealing and the authors' approaches are well-chosen and cutting-edge. The question is interesting and the approaches solid, although we find some of the claims over-interpreted, requiring further evidence and/or qualification. Specific points are listed below, but to summarize briefly: to qualify as a "state", we think that L2lag would deserve a more thorough characterization. Moreover, it remains puzzling why the effect appears largely limited to L2, especially in wild-type animals. This appears to contrast with the authors' claim of having demonstrated general modularity of C. elegans (larval) development. In addition, for daf-2, it is unclear how specific the effect is to the mutant allele employed. Finally, it seems to us that incomplete seam cell division is insufficient to explain the lag, several conditions appear to exhibit major differences in division timing but rather modest effects on L2lag occurrence or duration; this point would deserve clarification in the discussion.

Major points (for points involving experiments, we indicate whether essential, strongly recommended, or optional):

1. ESSENTIAL: The L2lag phase requires better characterization, at this point, it is not clear whether this is a developmental or a behavioral phenomenon. Do the worms still have a plug? Are they pumping? Are they moving? RECOMMENDED: Have the authors looked at other developmental events that can be timed; e.g., is Q-cell migration normal in daf-2?

2. Along the same lines, the claim that L2lag coincides with a pause in oscillatory gene expression requires more evidence and better presentation of available evidence. From the present analysis in Fig. 1H-J, it seems difficult to exclude an alternative scenario where the signal merely scales with a longer L2 in daf-2 mutants (i.e., taking longer to reach the peak and decline again, rather than exhibiting a lag/pause before increasing at normal pace). The authors must show the dynamics for the entire L2/oscillation period (i.e. going up an down) both in experimental time and scaled to the duration of each larva's L2 stage. RECOMMENDED: Their point would also be greatly strengthened by inclusion of another reporter, ideally expressed in another phase, to test whether the observation applies to a larger fraction of oscillating genes.

Panel H requires a better description - what are the individual dots? Can trajectories be discerned at the level of individual animals? It would be important to show some representative trajectories to understand the technical noise in the data and thus the quality of the fit. Why does the signal plateau - should the fit not involve an oscillatory/sinusoidal curve? In I), what are the dashed lines? We assume CI or a similar measure, but if so, it appears surprising that it would be so narrow given the variability of L2Lag - please comment. Is the left panel in J only a schematic or is the time scale on the x-axis missing?

3. ESSENTIAL: The fact that the lag appears to occur predominantly in L2 immediately suggests a connection to L2d/dauer. What happens in animals committed to dauer in different ways, and/or with other daf-2 mutant alleles; is there a correlation to the penetrance of Daf-c phenotype and/or larval stage extension? Is there something special about the e1370 allele used?

4. Fig. 2: According to the authors' model, daf-2-dependent insulin signaling can be either rescued by mutating the effector of the pathway, DAF-16 or the upstream phosphatase DAF-18. This implies a linear pathway where daf-16 mutation should recapitulate daf-18, but this is not the case - daf-18 is a more potent suppressor of L2lag, even in wild-type animals, pointing to a DAF-16-independent function. Moreover, in Fig. 2E, it appears that L2Lag behaves differently in wild type (fully suppressed by daf-18) and daf-2 (partially suppressed). Can the authors explain these observations?

5. ESSENTIAL: Fig. 3B: The authors' claim that lin-28 RNAi does not alter the "L2lag" is interesting, but have the authors confirmed that their RNAi treatment is effective? For instance, have they verified that they see some of the previously described heterochronic mutations (ideally the skipping of the symmetric seam cell division that is critical to their interpretation)? Is the fraction of wild type animal undergoing L2lag reduced as well? )? Given that wt and daf-2 appear to behave differently with regard to L2lag (duration, penetrance, daf-18-dependence), it would seem valuable to repeat these experiments (lin14 and lin-28 RNAi) on daf-2 mutant animals.

6. Fig 3C, D: We do not quite understand the rationale or interpretation of the lin-42 experiments. Is it clear that the results reflect a heterochronic and not a molting clock function of lin-42 (e.g, http://dx.doi.org/10.1016/j.cub.2011.10.054, https://doi.org/10.1038/s44318-025-00585-z)?

7. ESSENTIAL: Fig. 4: It appears that a control is missing here; daf-18 single mutant. Without it, we find it difficult to interpret the apparent acceleration of div1 and 2 in the double mutant vs. wild type, as also pointed out in ll. 216-217.

8. Line 208-215: The authors claim that div1 occurs at a similar time relative to hatching, while div 2 is also delayed in the daf-2 mutant, but to a larger extent than ecdysis 1. However, ecd1 is scored from hatch, while div2 from div 1 - which occurs ~5h after hatch, making for a substantially smaller denominator. Since the point that ecd1 and div2 are differentially affected is made more convincingly by Fig. 4B,D, we suggest omitting S2 and the related text; if the authors prefer to keep it, we would request to reference both events to the same landmark; either hatch or div1.

9. Ll. 220 - 222: We do not understand where/how Fig. 4E (and G) shows a correlation of the levels of desynchrony and the duration of L2lag. Where is desynchrony quantified and how? Does this refer to 4D and F respectively? If so and we eyeball the effects, it appears that div2 in Fig. 4D differs by ~2h between mutant and wt, but the lag increases by only 30 min in 4E - does this not contradict the authors' model that seam cell division needs to be completed to to initiate bona fide I2? The same points apply to Fig. 5H, and contrary to the phrasing in ll. 286-287, we find it surprising how modestly (if marginally statistically significant) HU treatment extends L2lag although it delay seam cell division substantially.

10. Along the same lines, the authors state (l.237) that in the V6 division of the daf-2; daf-16 double mutant occurs after ecdysis in 56% of the animals, and conclude by saying that the result is in line with a partial rescue of the L2lag phenotype. However, it seems to us the div2 relative to ecd1 shown in Fig S2D is similar to the one shown for daf-2; daf-18 in Fig 4D, and less than what wt shows in that figure. Please state those fractions, explain whether or not there is a significant difference and whether this is then consistent with the conclusion in the following sentence. We also suggest replotting the wt from Fig. 4 for reference with the mutants in S2D.

11. Fig. S2C, ll. 233-234: daf-2 mutation appears to mostly cause a reduction in DAF-16::GFP, not so much of a nuclear translocation. This seems unexpected, also by the authors' own preceding description of the pathway. Please explain.

12. Fig. 5A: This is a very nice experiment, but it lacks some technical description to fully understand it and its limitations: Is the assays stopped multiple times to extract individual worms or are many worms extracted at once? How long does it take to extract larvae, mount and watch, and were all animals manipulated and tested in parallel and from the same plate or in separate experiments etc.?

13. Fig. 5B, ll.259-267: The experiment lacks proper description - what genotype and how many animals were assayed? From discussion (ll. 352-354) we infer daf-2, but it is not clear. Nor can we understand the legend, please rephrase. What do the different lines represent? Animals with divisions not yet completed at ecdysis appear to have reduced luminescence signal, as predicted by the authors' model - but the drop appears moderate. Please reanalyze the data and take the 20% or 50% of animals with lowest luc expression at ecdysis and a) plot their luminescence vs. the other animals' for comparison and b) quantify fraction with undivided seam cells for each group.

14. We also infer from Fig 5B that animals with a lag phase also appear to have an extended M1 molt - is this correct? This is not obvious from Fig 1C and D - can the authors please comment? If this is a general feature, it would be helpful to mention it with introduction of Fig. 1, if not, the difference needs to be explained.

15. The authors show that HT115 as a food source accelerates seam cell division, but it seems that V6 cells still divide after ecdysis in daf-2 on this food - with point 9 and 10 above, this would seem to argue against the simple "seam cell division check point" model?! Please also reference literature showing accelerated seam cell division (or if a new discovery of the present study, state so.).

16. Fig 5I: The authors aim to show that L2lag is an independent mechanism that does not scale with larval stage duration by evaluating L2lag on the population level for different growth temperatures. OPTIONAL: They could strengthen this argument by evaluating it for single animals, ideally correlating the L2lag with timing in seam cell division (optional but highly interesting). OPTIONAL: Moreover, it would be useful to see the effect of temperature in daf-2 mutants, where the two timers are desynchronized (ditto).

17. The discussion needs to be improved. It seems unnecessary to have a long paragraph on the virtues of the luminescence assay (first paragraph of Discussion). More importantly, two pages of discussion on the "differential impact of IIS on developmental events" seem particularly excessive when the entire conclusion relies on only one perturbation (daf-2(e1370)) of IIS signaling. We find the last paragraph (ll. 369 - 378) particularly confusing as it appears to imply that insulin signaling is an integral component of L2lag generation or determination. This seems at odds with the remainder of the manuscript arguing that perturbed insulin signaling affects seam cell division more strongly than molting, necessitating the lag phase for realignment - i.e., IIS perturbation is a tool to uncover L2lag, not a mechanism controlling it.

18. To make a general claim of modularity of the system, we think stronger/additional data would be needed - going beyond L2 (where most of the current data was collected) and focusing on events that go beyond seam cell division and ecdysis. If these data are not available, we suggest strongly toning down, or omitting entirely, this part.

Minor points/typos/presentation:

1. Some bar charts seem to have a lot of overlapping data points, particularly around 0 (e.g. Fig. 1E) that end up being highly overplotted - perhaps a violin plot offers better representation of the data structure.

2. Line 41-42: consider rephrasing, currently sounds as if intermolt is part of molt.

3. Line 72-74: "intermolt" in its literal meaning is the time between two molts; hence, it is a bit unclear what is meant by "delaying the initiation of the following intermolt"

4. Line 84: "bocks" => "blocks"

5. Fig. 1G: Is this needed? The effect is not striking and there does not really seem to be a conclusion by the authors. Also the Table S1 referenced in the legend should probably be S2 and we could not find a File S1 (referenced also ll. 170-173).

6. L. 120: "Lags occurs" => "lag occurs, and later "larva stages" => "larval stages". Moreover, please recheck references for this sentence. References 16 and 18 are written in round brackets and it appears to us ref. 24 should be the ref. to cite here.

7. L. 125 and elsewhere: "concealed" => "contained"

8. Fig. 2A is not very accessible. A "split" figure where one panel shows the situation with active, one with inactive DAF-2 signalling would probably help. What are the grey blobs? Either label and explain all components or, preferred, simplify. Ll.154-156 refers to the interaction between DAF-18 and AGE-1, so AGE-1 should probably be labeled in the figure.

9. L. 144: "…phenotypes observed in daf-2 mutants as increased duration of the increased duration of the L2 stage, are absent in the double mutant daf-2(e1370);daf-16(mu86)". The authors showed in Fig 2C that the L2lag is only partially rescued, so the overall larval stage should still be longer?!

10. L. 146: "… show a duration of the second intermolt… (Fig 2B)". intermolt => Bona fide intermolt

11. Ll. 154-156: please state that DAF-18 is PTEN in worms

12. L. 205: "Using strains with the reporter matIs38…" - please indicate what this transgene exactly "reports" on; it appears to be a rather complex transgene array.

13. Fig 4A: micrographs are too small

14. L. 225: "pronounced that" => "pronounced than"

15. L. 263: "different" => "difference" and later "… between animals had already…" => "between animals that had already…"

16. Ll. 328-331: "a brake … at the level of molting" - since molting appears to occur at the normal time and for the normal duration, it is unclear that there is a specific brake on molting (rather than on intermolt).

17. L. 425: call-out should presumably be to Table S1

18. L. 461: "sticked" => "stuck"

19. Ll. 466-467: parenthesis make the sentence hard to read

20. Ll. 495-509: Luminescence recording: we are unsure how this was done. If signal was integrated over 1 min, the worm would be expected to have moved substantially, so how is the ROI then defined from the transmitted light image?

Rev. 3:

In this manuscript, the authors establish that developmental programs in C. elegans, such as seam cell division and ecdysis, are regulated independently and a loss of synchrony between these processes can lead to a subsequent delay in the molting program that allows resynchronization of development. This prevents a knock-on effect whereby all subsequent developmental larval stages could be adversely affected by earlier delays. This suggests a degree of developmental flexibility and the existence of mechanisms that allow coordination of developmental timing across different tissues and processes. These findings have important implications for understanding the regulation of development and are therefore likely to be appreciated both in the C. elegans field and beyond.

The authors use several innovative approaches to characterize developmental timing in individual animals. The analysis of cell division timing relative to molts is detailed and comprehensive. The use of the luciferase reporter strain is fundamental to the identification of the lag periods and is a novel application of this strain. This reporter has previously been validated by the authors as a marker of larval growth and the timing of molts. During the molts, luminescence is diminished due to the inability to ingest the luciferin substrate. In the current study, the authors determine that many animals have a delay in the restoration of the luminescent signal after ecdysis, despite completion of the molt, which they define as a lag phase. This lag phase occurs in a significant fraction of wild-type animals but is exacerbated in mutants that affect the insulin signaling pathway, as well as being influenced by nutritional status, with considerable inter-individual variability in lag duration.

My main question is whether this reporter is providing a readout of a fundamental biological process that underpins the observed lag period or whether we are observing an artificial, construct-specific phenomenon that happens to correlate with seam cell division and ecdysis? With respect to this question, is it possible to provide an orthogonal measure, independent of the luciferase reporter, that demonstrates the existence of the lag phase? For example, does the L2lag cause a delay in the resumption of growth at the level of the whole body?

The Psur-5::luc+::GFP transgene is an exogenous construct integrated into the C. elegans genome and the sur-5 promoter is described as constitutive. A previous study by the same authors showed that the molt does not affect luciferase protein expression in wild type worms. However, sur-5 gene expression does change during development, including during dauer formation, a process that is affected by changes in nutritional state and the daf-2 insulin signaling pathway. There also appear to be DAF-16 binding sites in the sur-5 promoter. Moreover sur-5 encodes a protein that, according to Wormbase, is predicted to enable acetoacetate-CoA ligase activity and be involved in a fatty acid metabolic process. Based on this, is it possible that sur-5 promoter activity is subject to nutritional regulation that affects luciferase expression after the molts in the various backgrounds? With this in mind, it would be useful to determine to what extent luciferase protein levels are affected by the daf-2 mutation, DAF-16 activity or nutritional state (OP50 vs HT115). Could the same lag phase be demonstrated with luciferase driven by different constitutive promoter?

The identity of the biological factor(s) that underpin the lag response remains an unanswered question in this study. Although their identification may ultimately be beyond the scope of this manuscript, there are several candidates that could be assessed fairly trivially. For example, on completion of the molt, does pharyngeal pumping restart immediately and, if so, is luciferin immediately available in vivo? Is there variability in the timing of the resumption of pumping or the efficiency of pumping that limits ingestion and / or bioavailability of luciferin? If the luciferase protein level is unchanged, and luciferin is bioavailable, does that mean that ATP availability is the rate-limiting factor? If so, does this suggest that the desychrony is fundamentally linked to ATP generation and therefore associated with mitochondrial function?

# Author comment: Author response to Decision Letter 2

**DOI:** 10.1371/journal.pbio.3003867.r003

31 Mar 2026

Attachment Submitted filename: Response to reviewers.pdf

# Peer review: Decision Letter 2

**DOI:** 10.1371/journal.pbio.3003867.r004

**Authors:** Ines Alvarez-Garcia

13 May 2026

Dear Dr Olmedo,

Thank you for your patience while we considered your revised manuscript entitled "Desynchrony between events triggers a compensatory delay during C. elegans development" for publication as a Research Article at PLOS Biology. This revised version of your manuscript has been evaluated by the PLOS Biology editors, the Academic Editor and the three original reviewers.

Based on the reviews (attached below), we are likely to accept this manuscript for publication, provided you satisfactorily address the remaining points raised by Reviewer 3. Please also make sure to address the data and other policy-related requests stated below my signature.

In addition, we would like you to consider a suggestion to improve the title:

"A desynchrony mechanism between events triggers a compensatory delay in C. elegans development"

As you address these items, please take this last chance to review your reference list to ensure that it is complete and correct. If you have cited papers that have been retracted, please include the rationale for doing so in the manuscript text, or remove these references and replace them with relevant current references. Any changes to the reference list should be mentioned in the cover letter that accompanies your revised manuscript.

In addition to these revisions, you may need to complete some formatting changes, which you will receive in a follow up email. A member of our team will be in touch with a set of requests shortly. If you do not receive a separate email within a few days, please assume that checks have been completed, and no additional changes are required.

We expect to receive your revised manuscript within two weeks.

To submit your revision, please go to https://www.editorialmanager.com/pbiology/ and log in as an Author. Click the link labelled 'Submissions Needing Revision' to find your submission record. Your revised submission must include the following:

- a cover letter that should detail your responses to any editorial requests, if applicable, and whether changes have been made to the reference list

- a Response to Reviewers file that provides a detailed response to the reviewers' comments (if applicable, if not applicable please do not delete your existing 'Response to Reviewers' file.)

- a track-changes file indicating any changes that you have made to the manuscript.

NOTE: If Supporting Information files are included with your article, note that these are not copyedited and will be published as they are submitted. Please ensure that these files are legible and of high quality (at least 300 dpi) in an easily accessible file format. For this reason, please be aware that any references listed in an SI file will not be indexed. For more information, see our Supporting Information guidelines:

https://journals.plos.org/plosbiology/s/supporting-information

*Published Peer Review History*

Please note that you may have the opportunity to make the peer review history publicly available. The record will include editor decision letters (with reviews) and your responses to reviewer comments. If eligible, we will contact you to opt in or out. Please see here for more details:

https://plos.org/published-peer-review-history/

*Press*

Should you, your institution's press office or the journal office choose to press release your paper, please ensure you have opted out of Early Article Posting on the submission form. We ask that you notify us as soon as possible if you or your institution is planning to press release the article.

*Protocols deposition*

To enhance the reproducibility of your results, we recommend that if applicable you deposit your laboratory protocols in protocols.io, where a protocol can be assigned its own identifier (DOI) such that it can be cited independently in the future. Additionally, PLOS ONE offers an option for publishing peer-reviewed Lab Protocol articles, which describe protocols hosted on protocols.io. Read more information on sharing protocols at https://plos.org/protocols?utm_medium=editorial-email&utm_source=authorletters&utm_campaign=protocols

Please do not hesitate to contact me should you have any questions.

Sincerely,

Ines

--

Ines Alvarez-Garcia, PhD

Senior Editor

PLOS Biology

ialvarez-garcia@plos.org

------------------------------------------------------------------------

DATA POLICY:

Thank you for submitting a data file. However, please note that we require all individual quantitative observations that underlie the data summarized in the following figures:

Fig. 1E-J; Fig. 2B-E; Fig. 3B, D; Fig. 4B, D, E, F; Fig. 5A-C, E, F, H, I; Fig. 6A-F, H; Fig. S1A-F; Fig. S2A, B; Fig. S3A-D, F-H; Fig. S4 and Fig. S5A-F

NOTE: the numerical data provided should include all replicates AND the way in which the plotted mean and errors were derived (it should not present only the mean/average values).

Please also ensure that figure legends in your manuscript include information on WHERE THE UNDERLYING DATA CAN BE FOUND.

Please ensure that your Data Statement in the submission system accurately describes where your data can be found.

------------------------------------------------------------------------

CODE POLICY

Per journal policy, if you have generated any custom code during the course of this investigation, please make it available without restrictions. Please ensure that the code is sufficiently well documented and reusable, and that your Data Statement in the Editorial Manager submission system accurately describes where your code can be found. More information on our Code Policy, what and how to share can be found here: https://journals.plos.org/plosbiology/s/code-availability

Please note that we cannot accept sole deposition of code in GitHub, as this could be changed after publication. However, you can archive this version of your publicly available GitHub code to Zenodo. Once you do this, it will generate a DOI number, which you will need to provide in the Data Accessibility Statement (you are welcome to also provide the GitHub access information). See the process for doing this here: https://docs.github.com/en/repositories/archiving-a-github-repository/referencing-and-citing-content

------------------------------------------------------------------------

Reviewers' comments

Rev. 1:

I am satisfied with the new experiments added and the authors' rebuttal successfully resolves my concerns. I have no further comments and recommend publication.

Rev. 2:

The authors have largely addressed our criticisms, and we appreciate the effort put into the revisions. What is currently missing is a (clear) description of the new figure panels 4E and 5I. We understand that some correlation is shown, but the underlying model, and the identity of the curved lines, are unclear. We are willing to take the authors' word that it supports their claim, but this should be clarified in the published version. The statement in ll. 314 - 318 is rather opaque, can the authors rephrase it to clarify what "was not as that from daf-18" means - and whether their data support or don't support their model? (Call-outs should also be corrected to Fig. S3 rather than S2, both for this entire paragraph and the subsequent one.)

Rev. 3:

Accept.

# Author comment: Author response to Decision Letter 3

**DOI:** 10.1371/journal.pbio.3003867.r005

29 May 2026

Attachment Submitted filename: Response to reviewers_2.pdf

# Editor report: Decision Letter 3

**DOI:** 10.1371/journal.pbio.3003867.r006

**Authors:** Ines Alvarez-Garcia

6 Jun 2026

Dear Dr Olmedo,

Thank you for the submission of your revised Research Article entitled "A desynchrony mechanism between events triggers a compensatory delay during C. elegans development" for publication in PLOS Biology. On behalf of my colleagues and the Academic Editor, Ursula Jakob, I am delighted to let you know that we can in principle accept your manuscript for publication, provided you address any remaining formatting and reporting issues. These will be detailed in an email you should receive within 2-3 business days from our colleagues in the journal operations team; no action is required from you until then. Please note that we will not be able to formally accept your manuscript and schedule it for publication until you have completed any requested changes.

Please take a minute to log into Editorial Manager at http://www.editorialmanager.com/pbiology/, click the "Update My Information" link at the top of the page, and update your user information to ensure an efficient production process.

PRESS

We frequently collaborate with press offices. If your institution or institutions have a press office, please notify them about your upcoming paper at this point, to enable them to help maximise its impact. If the press office is planning to promote your findings, we would be grateful if they could coordinate with biologypress@plos.org. If you have previously opted in to the early version process, we ask that you notify us immediately of any press plans so that we may opt out on your behalf.

We also ask that you take this opportunity to read our Embargo Policy regarding the discussion, promotion and media coverage of work that is yet to be published by PLOS. As your manuscript is not yet published, it is bound by the conditions of our Embargo Policy. Please be aware that this policy is in place both to ensure that any press coverage of your article is fully substantiated and to provide a direct link between such coverage and the published work. For full details of our Embargo Policy, please visit http://www.plos.org/about/media-inquiries/embargo-policy/.

Many congratulations and thanks again for choosing PLOS Biology for publication and supporting Open Access publishing. We look forward to publishing your study.

Sincerely,

Ines

--

Ines Alvarez-Garcia, PhD

Senior Editor

PLOS Biology

ialvarez-garcia@plos.org
